# Hydration-level-driven buffering effects on the compressibility of ion-exchanged mordenite

**DOI:** 10.1080/14686996.2025.2604928

**Published:** 2025-12-18

**Authors:** Soojin Lee, Hyunseung Lee, Jeongmin Kong, Dayeon An, Hyeonsu Kim, Pyosang Kim, Donghoon Seoung, Taeyeol Jeon, Katherine Armstrong, Sunki Kwon, Chung-Mo Lee, Huijeong Hwang, Yongmoon Lee

**Affiliations:** a Department of Geological Sciences, Pusan National University, Busan, Republic of Korea; b Department of Earth and Environmental Sciences, Chonnam National University, Gwangju, Republic of Korea; c Pohang Accelerator Laboratory, POSTECH, Pohang, Republic of Korea; d Advanced Light Source, Lawrence Berkeley National Laboratory, Berkeley, CA, USA; e Department of Earth and Planetary Sciences, University of California, Santa Cruz, CA, USA; f Institute for Future Earth Environment, Pusan National University, Busan, Republic of Korea; g School of Environment and Energy Engineering, Gwangju Institute of Science and Technology, Gwangju, Republic of Korea

**Keywords:** Mordenite, high-pressure, comparative compressibility, structural buffer effect, pressure-induced hydration

## Abstract

Understanding how large-pore zeolites respond to high-pressure conditions is essential for optimizing their structural stability and functional performance. In this study, we systematically investigated the compressibility and pressure-induced hydration (PIH) behavior of ion-exchanged mordenites using synchrotron X-ray powder diffraction under water-mediated conditions. The results reveal that the hydration level and spatial distribution of extra-framework cations (EFCs) at ambient conditions critically determine the initial number and arrangement of water molecules within the 12-membered ring (12MR) channels. Samples with weakly hydrated EFCs (e.g. Cs-MOR, Na-MOR) undergo a phase transition from ***C**mcm* to ***P**bnm* at about 1.6(1) GPa, because they fail to maintain the structural stability of the framework as compressed in water. In contrast, samples with EFCs strongly hydrated and uniformly distributed near the channel center (e.g. Sr-MOR, Eu-MOR) have lower compressibility, compared to cations aggregated near the channel wall (e.g. Pb-MOR, Cd-MOR). This study demonstrates that PIH acts as a structural buffer that stabilizes the framework by preventing pore collapse, thereby enhancing the compressibility in water. These findings underscore the critical role of the ambient EFC hydration state and PIH in governing the mechanical response of mordenite. The insights provide a basis for tailoring zeolite frameworks with optimized structural buffering effects for advanced industrial applications and geoscientific processes under extreme conditions.

## IMPACT STATEMENT

We systematically investigated structural evolution and compressibility of ion-exchanged mordenites under water-mediated high-pressure; intensely hydrated, centrally distributed cations (e.g. Sr- and Eu-MOR) facilitate higher levels of pressure-induced hydration.

## GRAPHICAL ABSTRACT

**Figure:**
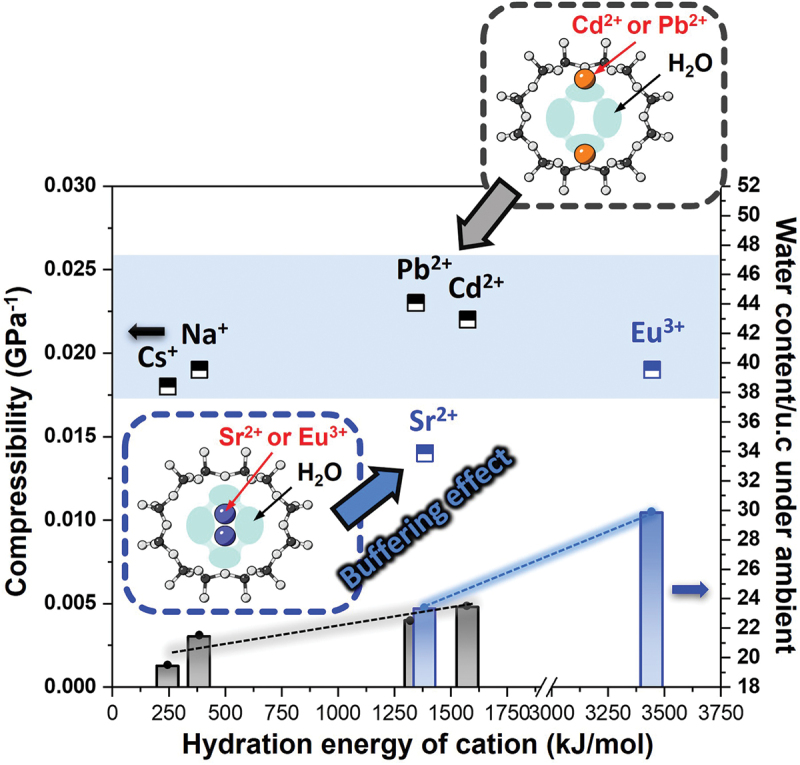

## 1. Introduction

Zeolites, as microporous materials, have been widely used in various industries due to their unique physical and chemical properties. While their thermal transformations, catalytic behavior, ion-exchange capabilities, and molecular sieving properties have been extensively investigated, the intrusion of pressure-induced guest molecules, and their interactions with the zeolite framework and extra-framework cations, remain relatively less well understood [1]. Understanding how different zeolite frameworks respond to high-pressure (*HP*) conditions is of great importance for their practical applications [2]. Pressure-induced compression can significantly alter pore geometries, increase the selective uptake of guest molecules, improve accessibility and diffusion to catalytic sites [2]. Suitable guest molecules and cations can fill the pores of the zeolite, they can even be adopted as ‘moulds’ for creating regular arrays of molecules to develop functional materials, such as luminescent materials obtained by the insertion of lanthanide arrays in the zeolite channels [3]. Moreover, hydrated zeolite under high-pressure strongly modifies the phase stability and the elastic properties [4]. This effect expands into hydrophobic, high silica zeolites coupled to nonwetting fluids, which are particularly adequate for mechanical energy storage and absorption for use as molecular springs, bumpers, and shock absorbers [4].

The location of water molecules and their interactions with the zeolite framework and exchangeable cations have been studied extensively, yet a clear understanding of the local structure and mobility of water and cations remains required [5]. Leeuw et al. employed classical energy minimization techniques to model the effects of hydration on the adsorption behaviour of the cations with zeolite A and CaNa-A. They demonstrate that the Na^+^ ions do not retain water molecules as strongly as the Ca^2+^ ions, which is why the Na^+^ ions are less stabilized in the β-cages [5]. Gómez-Álvarez and Calero et al. performed Grand Canonical Monte Carlo (GCMC) simulations to elucidate that the type of cation is more influential on the adsorption performance of water molecules than the amount of cation [6].

IR spectroscopy is highly sensitive to the short-range ordering and local structures in zeolites and, therefore, is suitable for in-situ monitoring of the structural changes between the crystalline and amorphous phases [7]. As reported in high-pressure IR studies by Huang et al., siliceous zeolite Y undergoes irreversible pressure-induced amorphization, whereas hydrated NaY shows reversible behavior. These hydrated cations act as so-called ‘non-deformable units’ and are located at the sites near the D6R (Double 6-rings), which may help the D6R units to resist the effects of pressure [7,8].

In particular, mordenite (MOR-type zeolite) crystallizes in the ***C**mcm* space group and has an ideal composition of Na_8_Al_8_Si_4__0_O_9__6_·24 H_2_O. Its framework consists of edge-sharing five-membered rings of tetrahedra that form chains along the *c*-axis [9]. These chains are arranged into sheets of six-membered rings parallel to the (100) plane and are linked by four-membered rings, producing two intersecting channel systems along the (001) direction: a large 12-membered ring channel (12MRc) and a highly compressed 8-membered ring channel (8MRc). These channels are interconnected by side pockets accessible through 8-membered rings and staggered by c/2 at their intersections [10]. The nature and spatial distribution of extra-framework cations (EFCs) within these channels strongly affect the compressibility and hydration behavior of the framework.

Several studies have investigated the thermal behavior of mordenite, showing that during dehydration, cations migrate to new sites to achieve energetically favorable configurations, and the main deformation of the Si/Al framework occurs as a change in the ellipticity of the channels [11–15]. In situ *HP* synchrotron X-ray diffraction experiments have demonstrated that the elastic behavior of mordenite is highly anisotropic, with channels compressed differently along specific crystallographic directions [16–18]. Furthermore, extra water molecules that penetrate the pore system under *HP* conditions can act as structural fillers, mitigate framework collapse and modify the overall compressibility [18]. Recent PIH studies on small-pore zeolites have highlighted the increasing interest in how high-pressure conditions affect hydration and structural stability [19]. Although pressure-induced hydration has been reported for certain small-pore zeolites, systematic studies comparing the high-pressure structural evolution and PIH mechanisms of large-pore zeolites like mordenite with a variety of exchangeable cations are still lacking.

Understanding how different cations – especially spanning monovalent, divalent, and trivalent states – influence the capacity of mordenite to accommodate additional water under *HP* conditions is essential for clarifying the fundamental structure – property relationships of large-pore zeolites. Therefore, the aims of this study are: (1) to investigate the high-pressure structural evolution of ion-exchanged mordenites by means of synchrotron X-ray powder diffraction using water as a penetrating pressure-transmitting medium; (2) to examine the capacity of ion-exchanged mordenites to accommodate additional water molecules within the pore system under pressure; and (3) to clarify how the nature and spatial distribution of different cations affect compressibility and hydration mechanisms in large-pore zeolites. Such insights are crucial not only for understanding the fundamental behavior of zeolites but also for optimizing their application in extreme industrial processes and deep geological storage systems. These findings provide valuable implications for catalytic processes under extreme conditions and for water – rock interactions in deep subduction zones and geological CO_2_ storage.

## 2. Experimental details

### 2.1. Sample preparation

Na-MOR was used as the starting material for other exchanged mordenites. The preparation and characterization of Cs-, Na-, Pb-, Sr-, and Cd-MOR were described in detail by Lee et al. [20], and Eu-MOR was obtained using a Eu(NO_3_)_3_·5 H_2_O (from Sigma-Aldrich) solution following the same method. The elemental analysis of Eu-MOR was performed by an energy-dispersive X-ray spectrometer (EDS, SUPRA25, Oxford Instruments) operating at an accelerating voltage of 15 kV at Pusan National University, Korea. The ion-exchange rate of Eu^3+^ in Na-MOR was approximately 85%. Thermogravimetric analysis (TGA, TGA-50, Shimadzu) was conducted at Gwangju Institute of Science and Technology, Korea, to determine the H_2_O content in the pores by heating the sample to 800°C at a rate of 10 °C/min under a nitrogen atmosphere (Figure S1). The chemical composition of Eu-MOR was derived from the EDS method and TGA results, as summarized in Table S1.

### 2.2. High-pressure experiments

A symmetric-type diamond anvil cell (DAC, Beijing Scistar Technology Co. LTD., China) was used for the *HP* experiments at room temperature, equipped with a pair of type-Ia anvils with a culet diameter of 700 μm and tungsten-carbide seats [21]. A stainless steel gasket, 250 μm in thickness, was pre-indented to a thickness of ~80 μm and then drilled a micro-hole (sample chamber, diameter of ~350 μm) by electro-spark erosion. A sample was placed in the gasket hole together with ruby spheres (~20 μm) for pressure measurements. The pressure of the sample was measured by detecting the shift in the R1 emission line [22,23], and the estimated error in the pressure values is 0.1 GPa. We calculated pressure using the following equations:

$$P=19.04/7.665\left\{{\left[1+\left(\Delta \lambda /\lambda_{0}\right)\right]}^{7.665}-1\right\}$$

where *P* is the pressure in megabars, *λ*_*0*_ is the initial wavelength of the ruby R1 line, and *Δλ* represents the wavelength shift from λ_0_ [24]. Deionized water as a penetrating pressure-transmitting media (PTM), and silicone oil (AP150 from Sigma-Aldrich) as a non-penetrating PTM were used for *HP* experiments on cation-exchanged mordenites. All ambient conditions (*P*_*amb*_) data were collected from the dry powder sample loaded into the DAC. In the water *HP* experiments, water was added to the sample chamber, and second ambient condition data (termed as ‘wet’ condition, *P*_*wet*_) were taken using this wet sample. In the silicone oil *HP* experiments, the DAC was sealed to the first pressure point after being loaded. The pressure increased from *P*_amb_ up to 5.0(1) GPa, with Δ*P* increments of 0.5(1)−1.0(1) GPa. The sample was equilibrated for about 10 min in the DAC at each measured pressure. A final data set was measured after the pressure was released.

### 2.3. Synchrotron X-ray powder diffraction

High-pressure X-ray powder diffraction experiments were performed at beamlines 3D (XRS), 5A (MS-XRS), and 9A (U-SAXS) at the Pohang Light Source II (PLS II) in South Korea, and at beamline 12.2.2 (Diffraction Under Non-Ambient Conditions) at the Advanced Light Source (ALS), Lawrence Berkeley National Laboratory in the U.S.A.. At the PAL 3D beamline, the primary white beam from the bending magnet was monochromatized (0.6884(1) Å) using a water-cooling Si (111) double crystal monochromator (DCM) and focused by a toroidal mirror. At the PAL 5A beamline, the X-ray beam from the in-vacuum undulator insertion device was monochromatized (0.6927(1) Å) by a Si (111) DCM and focused by a pair of vertical and horizontal focusing mirrors. In both beamlines, a two-dimensional MAR345 imaging plate detector (100 μm pixel resolution, MarXperts) was positioned at 348 mm and 285 mm for XRD measurements. At the PAL 9A beamline, for a wide-angle X-ray diffraction experiment, the X-ray from the in-vacuum undulator was monochromatized (0.6268(1) Å) using a DCM equipped with Si (111) and Si (311) crystals and focused by Kirkpatrick-Baez (KB) mirrors. A 2D CCD MX170-HS detector (44 μm pixel resolution, Rayonix L.L.C.) was used to collect diffraction data at a distance of 241 mm. At the ALS 12.2.2 beamline, monochromatic X-ray from the superconducting bending magnets with a wavelength of 0.5327(1) Å and Pilatus 1 M detector (179 μm pixel resolution, DECTRIS) were used to collect diffraction data at a distance of 219 mm from the sample [25]. All the diffraction patterns were integrated using the DIOPTAS software [26]. The sample-to-detector distance and detector tilt, based on the direct beam, were refined based on a CeO_2_ (NIST SRM 674b) powder diffraction pattern analyzed with DIOPTAS [27].

### 2.4. Structural analysis by Rietveld refinement

Pressure-dependent changes in the unit-cell constants were determined by whole pattern fitting using the GSAS program suite [28–30]. The background curve was fitted by a Chebyshev polynomial with 36 coefficients, and the pseudo-Voigt profile function was used to model the observed Bragg reflection [31]. The structural models at selected pressure conditions were established by Rietveld methods [32]. The tetrahedral site was assumed to be statistically occupied by Si and Al atoms. The result of the chemical analysis was then used to set the Si/Al ratio to 0.86/0.14, corresponding to the disordered distribution of Si and Al atoms. Soft constraints on the T-O (T = Si, Al) and O-O interatomic distances of the tetrahedra were applied to keep the framework geometry: the distances between Si(Al)-O and O-O were restrained to target values of 1.638 ± 0.001 and 2.676 ± 0.005 Å, respectively [33]. To minimize the number of variables, isotropic displacement factors (*U*_iso_) were refined by grouping the framework tetrahedral atoms, the framework oxygen atoms, the extra-framework species respectively. The amounts of water molecules in the unit cell were determined by using the result of Rietveld refinement with OW1, OW2, OW3, and OW4 multiplicities and occupancies. The distributions of the extra-framework cations in the channel were based on residual electron density maps from difference Fourier synthesis. The final convergence of the refinement was achieved by simultaneously adjusting all background and profile parameters, scale factors, lattice constants, 2θ zero, and atomic positional and thermal displacement parameters [34]. The information on the final refined models is summarized in Figure S5 and Table S2.

## 3. Results and discussion

### 3.1. Comparative compressibility of mordenites with different exchangeable cations

**Figure 1.**
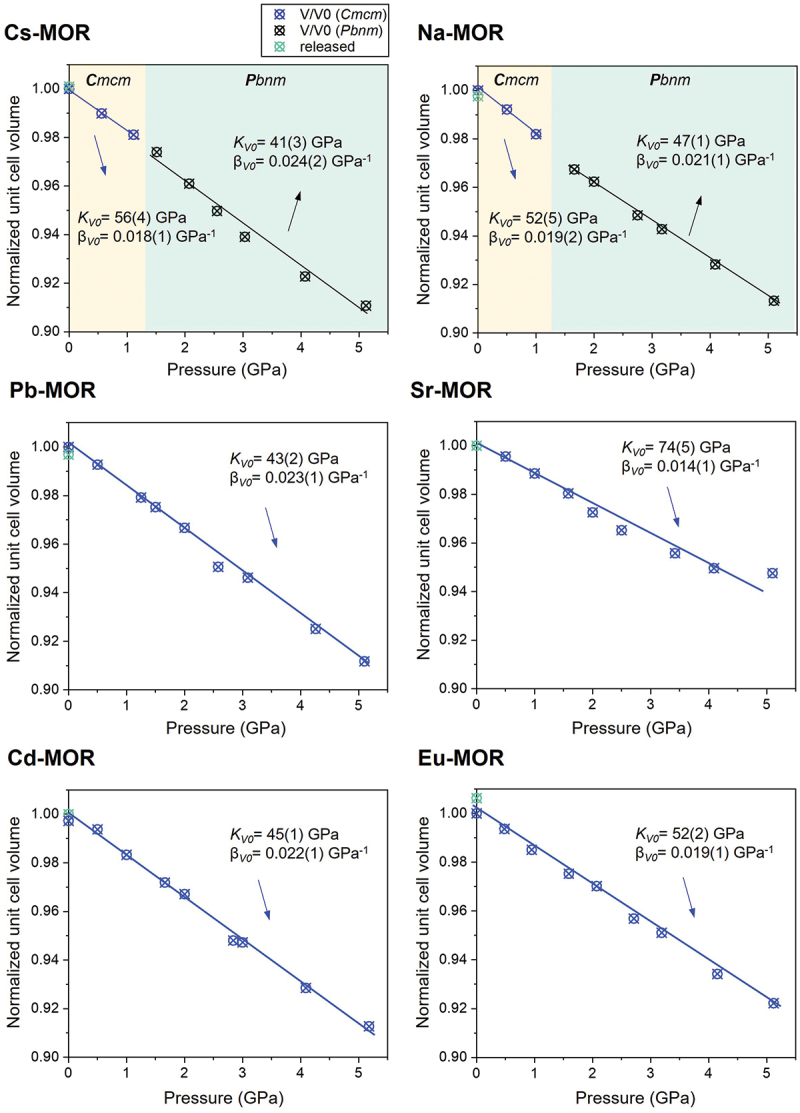
Normalized unit-cell volume of ion-exchanged mordenite (Cs-, Na-, Pb-, Sr-, Cd-, and Eu-MOR) compressed in water as a function of pressure. The experimental *P-V* data were fitted with a truncated second-order Birch-Murnaghan equation of state [35–37] using the EoS-Fit7 program [39]. For *V*, the esd values are smaller than the size of the symbols.

**Figure 2.**
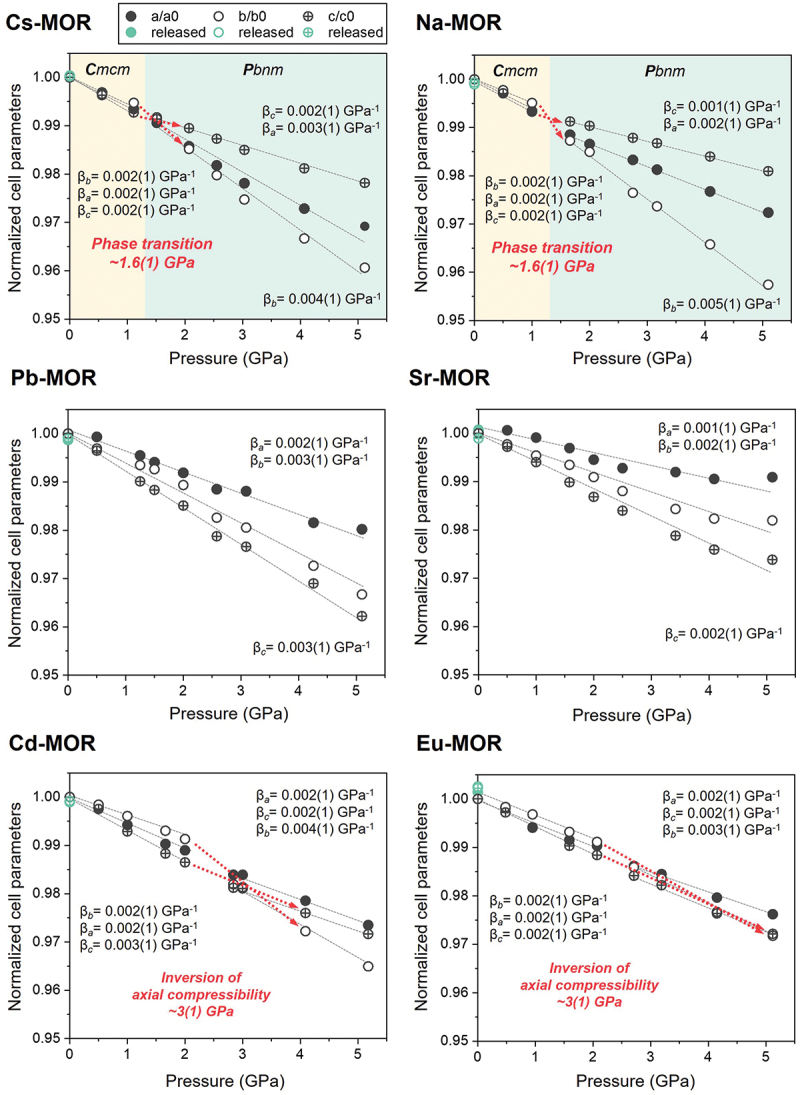
Normalized unit-cell parameters of ion-exchanged mordenite (Cs-, Na-, Pb-, Sr-, Cd-, and Eu-MOR) compressed in water as a function of pressure. The axial compressibility was calculated using ‘linearized’ equations of state, substituting the cube of a lattice parameter (*a*^*3*^, *b*^*3*^, and *c*^*3*^) for the volume in the equations [37]. For *a*, *b*, and *c*, the esd values are smaller than the size of the symbols.

To investigate the compressibility of cation-exchanged mordenites in water, the experimental *P-V* data were fitted with a truncated second-order Birch-Murnaghan equation of state [35–38] using the EoS-Fit7 program [39] (Figures 1 and 2). In the *HP* water experiment below 1.0(1) GPa, the refined EoS parameters for Na-MOR are: *K*_*V0*_ = 52(5) GPa, β_*V0*_ (1/ *K*_*V0*_) = 0.019(2) GPa^−1^, and *K*_*V*_’ = 4.0 (fixed) (Figure 1). The axial compressibility was calculated using ‘linearized’ equations of state, substituting the cube of a lattice parameter (*a*^*3*^, *b*^*3*^, and *c*^*3*^) for the volume in the equations [37] (Figure 2).

Zeolites generally exhibit significantly less compressibility in water because water molecules (~2.65 Å) can penetrate the zeolite pores under pressure [40]. The compressibility (β) of the remaining cation-exchanged mordenites is in the range 0.018(1) to 0.024(2) GPa^−1^, except for Sr-MOR (Figure 1). Notably, only monovalent cation forms (Cs- and Na-MOR) undergo a phase transition from ***C**mcm* to ***Pbnm*** at about 1.6 (1) GPa. Below the transition pressure, the compressibility of both Cs- and Na-MOR is relatively low compared to values above 1.6(1) GPa, due to a substantial penetration of water molecules. As pressure increases, the weakly hydrated cations (Cs and Na ions, in Table 1) fail to maintain the structural stability of the framework, resulting in a phase transition above 1.6(1) GPa. According to previous reports, a very high-volume compressibility [*K*_*V0*_ = 25(2) GPa, β_*V0*_ (1/ *K*_*V0*_) = 0.040(3) GPa^−1^, and *K*_*V*_’ = 2.0(3)], coupled with a remarkable elastic anisotropy (β_*b*_>> β_*c*_ > β_*a*_) was observed [18]. Also, a similar transition occurred in Na-mordenite compressed in an anhydrous 4:1 methanol:ethanol mixture between 1.68(7) and 2.70(8) GPa [18]. These results may be attributed to the penetration of molecules into the pores under pressure, regardless of molecular species; however, the mechanism remains unclear. The significantly lower compressibility for Sr-MOR compared to other mordenites is likely due to the hydration effects of the cations within the 12MR channels. The hydrated Sr^2+^ ions (hydration E: −1,385(5) kJ/mol) can almost entirely occupy the 12MR channels, thereby reinforcing the framework against pressure-induced deformation. As reported by Cho and Choi et al. [41], strongly hydrated Sr^2+^ ions (ionic radius: ~8.2 Å) are much larger than the micropore openings of most zeolites. The presence of large pores (e.g. 12MR) provides a significant structural advantage for achieving fast ion-exchange kinetics. Consequently, the mordenite framework with the 12MR channels exhibits greater resistance to compression when exchanged with Sr^2+^ than with other cations.

**Table 1.** The standard molar Gibbs free energy of hydration of cations at 298.15 K [51].

Cation	Cs^+^	Na^+^	Pb^2+^	Sr^2+^	Cd^2+^	Eu^3+^
Hydration energy (kJ/mol)	−245(5)	−385(20)	−1,345(80)	−1,385(5)	−1,575(18)	−3,350(10)

Cs- and Na-MOR show the least compression along the *b*-axis (β_*b*_ = 0.002(1) GPa^−1^) below the transition pressure, whereas compression along the *b*-axis (β_*b*_ = 0.004(1) − 0.005(1) GPa^−1^) become dominant above the transition pressure (Figure 2 and Table 2). Due to the continuous insertion of water molecules through the 8MR and 12MR channels parallel to the *c*-axis, they resist compression along the *b*-axis, which is the most readily compressible direction. However, mordenites with monovalent cation that are unable to maintain the framework effectively undergo a phase transition above 1.6(1) GPa, accompanied by compression along the *b*-axis.

**Table 2.** Bulk modulus and axial compressibility of ion-exchanged mordenite compressed in water.

Axial compressibility (GPa^−1^)		PTM (Water)					
		Cs-MOR	Na-MOR	Pb-MOR	Sr-MOR	Cd-MOR	Eu-MOR
β_a_	below	0.002(1)	0.002(1)			0.002(1)	0.002(1)
	above	0.003(1)	0.002(1)			0.002(1)	0.002(1)
	total			0.002(1)	0.001(1)		
β_b_	below	0.002(1)	0.002(1)			0.002(1)	0.002(1)
	above	0.004(1)	0.005(1)			0.004(1)	0.003(1)
	total			0.003(1)	0.002(1)		
β_c_	below	0.002(1)	0.002(1)			0.003(1)	0.002(1)
	above	0.002(1)	0.001(1)			0.002(1)	0.002(1)
	total			0.003(1)	0.002(1)		
Bulk modulus (GPa)	below	56(4)	52(5)				
	above	41(3)	47(1)				
	total			43(2)	74(5)	45(1)	52(2)

The data were fitted separately below and above 1.0(1) GPa for Cs- and Na-MOR, and 2.5(1) GPa for Cd- and Eu-MOR, respectively.

The axial compressibility of Pb- and Sr-MOR consistently decreases in the order of β_*c*_ *>* β_*b*_ *>* β_*a*_, with no reversals observed. The lower resistance to compression along the *c*-axis, compared to the *b*-axis, is attributed to the insertion of water molecules into the side-pocket parallel to this direction, which had a slightly more predominant effect. For Cd- and Eu-MOR, the compressibility of the *a*-, *b-*, and *c-*axes is nearly similar, with minimal water penetrating the pores. Below 2.0(1) GPa, the *b*-axes show the least compression (β_*b*_ = 0.002(1) GPa^−1^); however, above 4.0(1) GPa, it becomes the most compressible axis (β_*b*_ = 0.004(1) GPa^−1^). A detailed description of the pressure-induced hydration is provided in Structural refinement of exchanged MORs under Pwet and HP conditions.

To elucidate the anisotropic behavior of cation-exchanged mordenites under different PTM, compression experiments were conducted using silicone oil as a non-penetrating PTM under the same conditions as the water experiment, which served as the reference. The refined parameters for Na-MOR are *K*_*V0*_ = 25(1) GPa, β_*V0*_ (1/ *K*_*V0*_) = 0.040(1) GPa^−1^, and *K*_*V*_’ = 4.0 (fixed) (Figure S3). Linear compressibility along each crystallographic axis for Na-MOR are: β*ₐ* =0.003(1) GPa^− 1^, β_*b*_ = 0.006(1) GPa^− 1^, and β_*c*_ = 0.004(1) GPa^− 1^. These results reveal an anisotropic compressibility trend of β_*b*_ > β_*c*_ > β_*a*_, consistent with previous studies [16,18,42].

### 3.2. Structural refinement of exchanged MORs under ambient conditions

**Figure 3.**
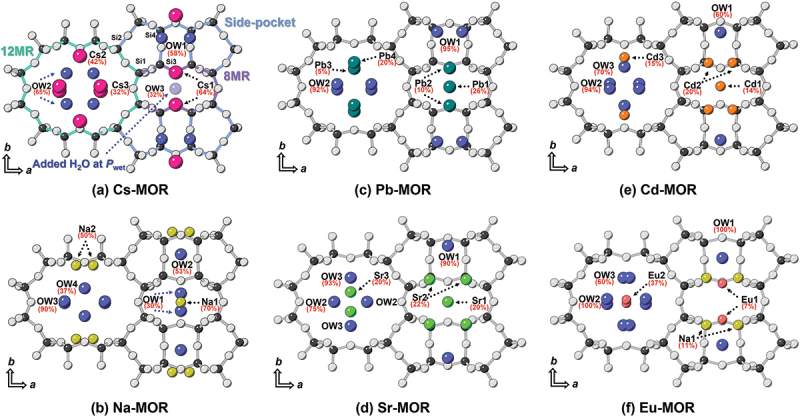
Representation of ion-exchanged mordenite structures ((a) Cs-, (b) Na-, (c) Pb-, (d) Sr-, (e) Cd-, and (f) Eu-MOR) at ambient conditions, viewed along the (001) direction. The distribution of extra-framework species shows no significant variation up to 1.0(2) GPa; however, in Cs-MOR, additional water molecules are introduced under wet conditions and are illustrated in light blue. Black circles denote the disordered distribution of Si(Al) atoms, and grey circles represent O atoms in the framework. Blue, magenta, yellow, dark green, bright green, orange, and red circles represent H_2_O molecules and the cations Cs^+^, Na^+^, Pb^2+^, Sr^2+^, Cd^2+^, and Eu^3+^, respectively.

The levels of hydration of cation-exchanged mordenites up to 1.0(3) GPa, below the solidification pressure of iceVI [43], were characterized by Rietveld refinement and are discussed here in correlation with the intrinsic properties of the exchangeable cations and their spatial distribution within the framework. Under ambient conditions, Cs^+^ ions prefer to locate near the framework due to their high charge density and are partially occupied at the Cs3 site, which may be occupied by either cations or water molecules in the large 12-membered ring (12MR) channels [44] (Figure 3). The water molecules in Cs-MOR are found within the side-pocket, and 12MR, while Na-MOR exhibits a more dispersed distribution, occupying the 8-membered rings (8MR), side pockets, and 12MR. The monovalent cation-exchanged Cs-MOR and Na-MOR contain 19.4 and 21.4 water molecules per 96 framework oxygens, respectively. Since the number of divalent cations is smaller, more sites in the cavities are available for water than in the case of monovalent cations [45,46]. Thus, divalent cations hydrate more strongly than monovalent ions of comparable radius [45]. Additionally, the spatial distribution of cations within the 12MR channels plays a crucial role in determining the water content. Pb^2+^ cations in Pb-MOR show a strong affinity for the electron cloud of the framework oxygen atoms because they have higher electronegativity than the framework cations [47], which positions them close to the walls of the 12MR channels. In the case of Sr-MOR and Eu-MOR, the structural environments within the 12MR channels are favorable for coordination with water molecules. In Sr-MOR, the Sr^2+^ cations located within the 12MR channels are distributed along the direction parallel to the *b*-axis and strongly hydrated with surrounding water molecules. Similarly, the Eu^3+^ cations are situated at the center of the 12MR channels, providing optimal geometry for coordination with water molecules and resulting in the highest water content among all the samples. The divalent cation-exchanged Pb-, Sr-, and Cd-MORs correspond to 22.4, 23.3, and 23.4 water molecules per 96 framework oxygens, respectively, while the trivalent cation-exchanged Eu-MOR corresponds to 29.8 under ambient conditions. These results indicate that the ambient hydration level and the location of EFCs within the 12MR channels play a crucial role in determining how many water molecules are accommodated and how they are distributed. This initial arrangement strongly influences the capacity for PIH during compression, effectively buffering the framework against collapse.

### 3.3. Structural refinement of exchanged MORs under P_wet_ and HP conditions

Since zeolites readily absorb water upon contact at room temperature and pressure, the *P*_*wet*_ data obtained under ambient conditions were compared with the high-pressure data to investigate PIH. Under *P*_*wet*_ conditions, changes in the occupancy of water molecules and the unit-cell volume were observed. Although no detectable shift in ruby fluorescence was found, the possibility of hydration induced by the anvils compressing the water-saturated sample cannot be excluded. The molecular diameter of water (~2.65 Å) is small enough to penetrate both the side-pocket (3.4 ×4.8 Å) and 12-membered ring channels (6.5 ×7.0 Å) of the MOR framework [48], which could lead to additional intrusion of water molecules into the pores. The potential water sites for this intrusion are illustrated as blue circles in Figure 3, and their distribution is in good agreement with reported by Maurin et al. [49].

**Figure 4.**
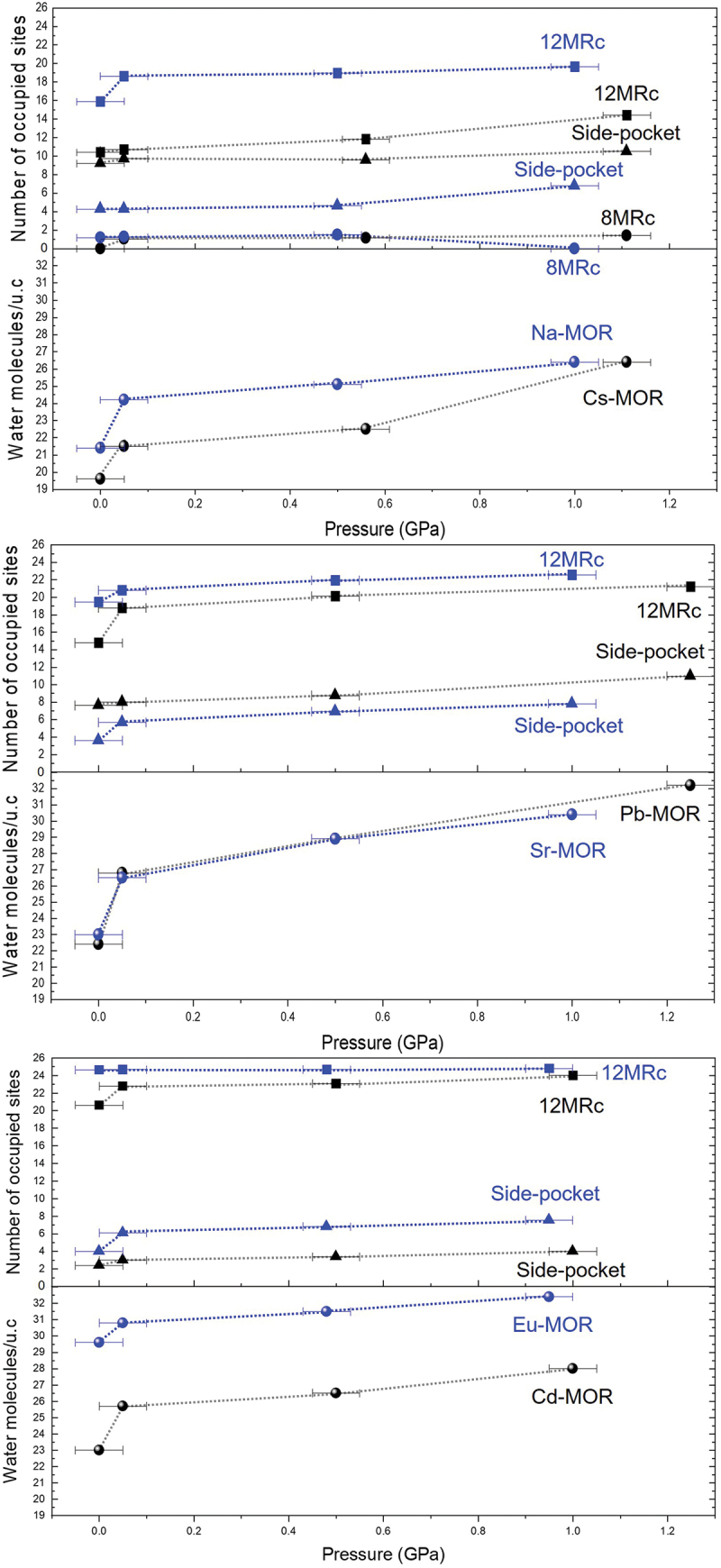
Changes in the number of water molecules intruding into the channels and in the total water content per unit-cell with increasing pressure. The data are plotted as three distinct groups classified based on their hydration energy values.

**Figure 5.**
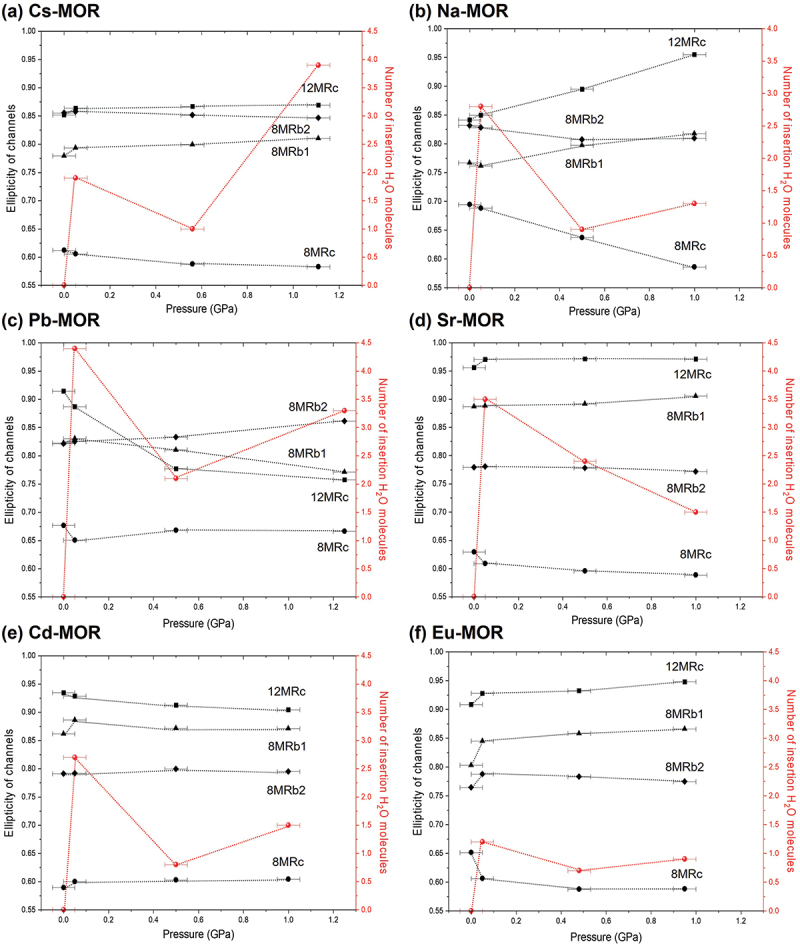
Ellipticity variations of the side-pocket, 8MRc, and 12MRc channels as a function of pressure. The number of water molecules intruding at each pressure is represented by red spheres. The side-pocket is divided into two components: 8MRb1 along the 8MRc direction and 8MRb2 along the 12MRc direction. Channel ellipticity (ε = short-axis/long-axis).

In Cs-MOR under *P*_*wet*_ conditions, the OW3 site is added to the center of the 8MRc, and the number of water molecules per 96 framework oxygen atoms increases from 19.6 to 21.5 (Figure 4). Up to 1.1 (1) GPa, the water content gradually increases in both the 8MRc and side-pocket channels, while a large amount of water mainly infiltrates into the 12MRc channels, resulting in a total water content of 26.4. The substantial intrusion of water under pressure contributes to retaining or increasing the ellipticity (ε, S/L) of the 12MRc channels. Consequently, the ellipticity of the 12MRc is preserved even above 1.1(1) GPa (Figure 5), which is consistent with the trend in axial compressibility, as described in Comparative compressibility of mordenites with different exchangeable cations. In Na-MOR, water molecules mainly enter the side-pocket and 12MRc, and the total number of water molecules per unit-cell increases from 21.4 to 26.4. Above 0.5(1) GPa, the OW1 site located at the center of the 8MRc migrates toward the side-pocket, resulting in an increase in the ellipticity of the 8MRc and a decrease in that of the 8MRb1. This migration of water may be attributed to the low hydration energy of the Na^+^ ions and to the side-pocket serving as a diffusion pathway for water within the framework. In the cases of Pb- and Sr-MOR, the spatial distribution of cations within the 12MRc channels clearly demonstrates their influence on the ellipticity of the channels compressed in water. The strong interaction between Pb^2+^ ions and the framework oxygen atoms within the 12MRc restricts their hydration by surrounding water molecules. Although the additional insertion of water molecules into what was the original Pb3 site under *P*_*wet*_ conditions, and the total number of water molecules per unit-cell increases from 22.4 to 32.2 at 1.2(1) GPa, the ellipticity of the 12MRc significantly increases as the water molecules gradually concentrate into the center of 12MRc with pressure. The intrusion of more water molecules than other divalent-cation forms above 1.0 GPa is likely due to pressure errors. In contrast, the Sr^2+^ ions are firmly coordinated to surrounding water molecules (OW2 and OW3 sites); as a result, the ellipticity of the channels is largely preserved up to 1.0 (1) GPa, with a total water content of 30.4. Specifically, the shape of 12MRc remained nearly circular under pressure as shown in Figure 5, which may have contributed to its enhanced structural rigidity.

A mechanism similar to that observed in Pb-MOR is also evident in Cd-MOR, where Cd^2+^ ions are positioned close to the framework atoms. This configuration results in a increase in the ellipticity of the 12MRc, despite the rise in the number of water molecules from 23 to 28 at 1.0(1) GPa. In Eu-MOR, Eu^3+^ ions are located at the center of the 12MRc and form a strong hydration shell (hydration E: −3,350(10) kJ/mol) coordinated by the OW2 and OW3 sites. This rigid hydration sphere reinforces the 12MRc channels under compression, which transform into a nearly circular shape; consequently, the 8MRc channels parallel to the crystallographic axis become increasingly elliptical in response. The water molecules preferentially penetrate the side-pockets, and the number of water molecules per unit-cell increases from 29.6 to 32.4 up to 1.0(1) GPa.

Although both Sr^2+^ and Eu^3+^ ions have high hydration energies and display similar hydration environments within the 12MRc, the compressibility of Eu-MOR (β = 0.019(1) GPa^−1^) is slightly higher than that of Sr-MOR (β = 0.014(1) GPa^−1^). This may be associated with the lower cation sites and significant restrictions on the additional intrusion of water molecules into the 12MR due to Eu^3+^, which is composed of hard hydration shells. Eu^3+^ in the 12MR was found to be 8-fold coordinated with H_2_O at an average Eu^3+^-O(H_2_O) distance of 2.33(2) Å at ambient condition, which is comparable to the EXAFS results (CN = 8.6, 2.43 Å) reported for Eu^3+^ exchanged zeolite Y [50].

Importantly, these observations suggest that the initial hydration level and spatial distribution of extra-framework cations (EFCs) under ambient conditions set the stage for how the framework accommodates compression. When the cations and water molecules are favorably arranged, they can promote more effective pressure-induced hydration (PIH), which acts as a structural buffer that mitigates framework collapse and results in a lower compressibility[51].

## 4. Conclusions

This study demonstrates that the structural evolution and compressibility of ion-exchanged mordenites under water-mediated high-pressure conditions are strongly influenced by the hydration level and spatial distribution of extra-framework cations (EFCs). Rietveld refinements confirmed that, at ambient conditions, the type and arrangement of EFCs govern the initial number and positioning of both cations and water molecules within the 12MR channels. This ambient structural state directly determines the capacity for pressure-induced hydration (PIH) during compression. The results reveal that cations with stronger hydration energies and more uniform distribution near the channel center (e.g. Sr- and Eu-MOR) resist compression with water. The additional water molecules act as a structural buffer that stabilizes the pore system and suppresses framework collapse, resulting in significantly reduced compressibility compared to samples with weaker ambient hydration levels and EFCs clustered near the channel wall (e.g. Pb- and Cd-MOR). Overall, this study highlights that the ambient EFC hydration level and the resulting cation – water arrangement critically control the extent of PIH achievable under high-pressure conditions. This PIH mechanism functions as an effective buffer that directly reinforces the framework’s mechanical stability. These insights provide a basis for understanding how the interplay between ion exchange, hydration behavior, and channel geometry can be exploited to design zeolites with tailored mechanical and adsorption properties for advanced industrial processes and geoscientific applications under extreme conditions.

## Supplementary Material

Supplemental Material
