# Supplementary material for: Hydration-level-driven buffering effects on the compressibility of ion-exchanged mordenite
Source: Sci Technol Adv Mater. 2025 Dec 18;27(1):2604928. doi: 10.1080/14686996.2025.2604928 (PMC12865833; doi:10.1080/14686996.2025.2604928)
Supplement: Supplemental Material [file TSTA_A_2604928_SM8520.docx]

**Supplementary material**

Hydration-Level-Driven Buffering Effects on the Compressibility of Ion-Exchanged Mordenite

Soojin Lee, Hyunseung Lee, Jeongmin Kong, Dayeon An, Hyeonsu Kim, Pyosang Kim, Donghoon Seoung, Taeyeol Jeon, Katherine Armstrong, Sunki Kwon, Chung-Mo Lee, Huijeong Hwang^*^ and Yongmoon Lee^*^

* Corresponding authors

E-mail: [lym1229@pusan.ac.kr](mailto:lym1229@pusan.ac.kr) and [huijeonghwang@gist.ac.kr](mailto:huijeonghwang@gist.ac.kr)


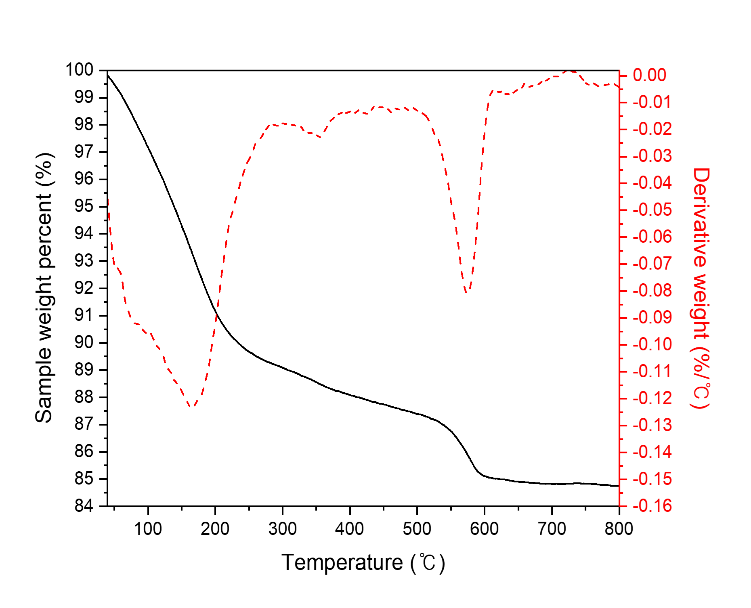


**Figure S1.** TG-DTG curves for Eu-mordenite.


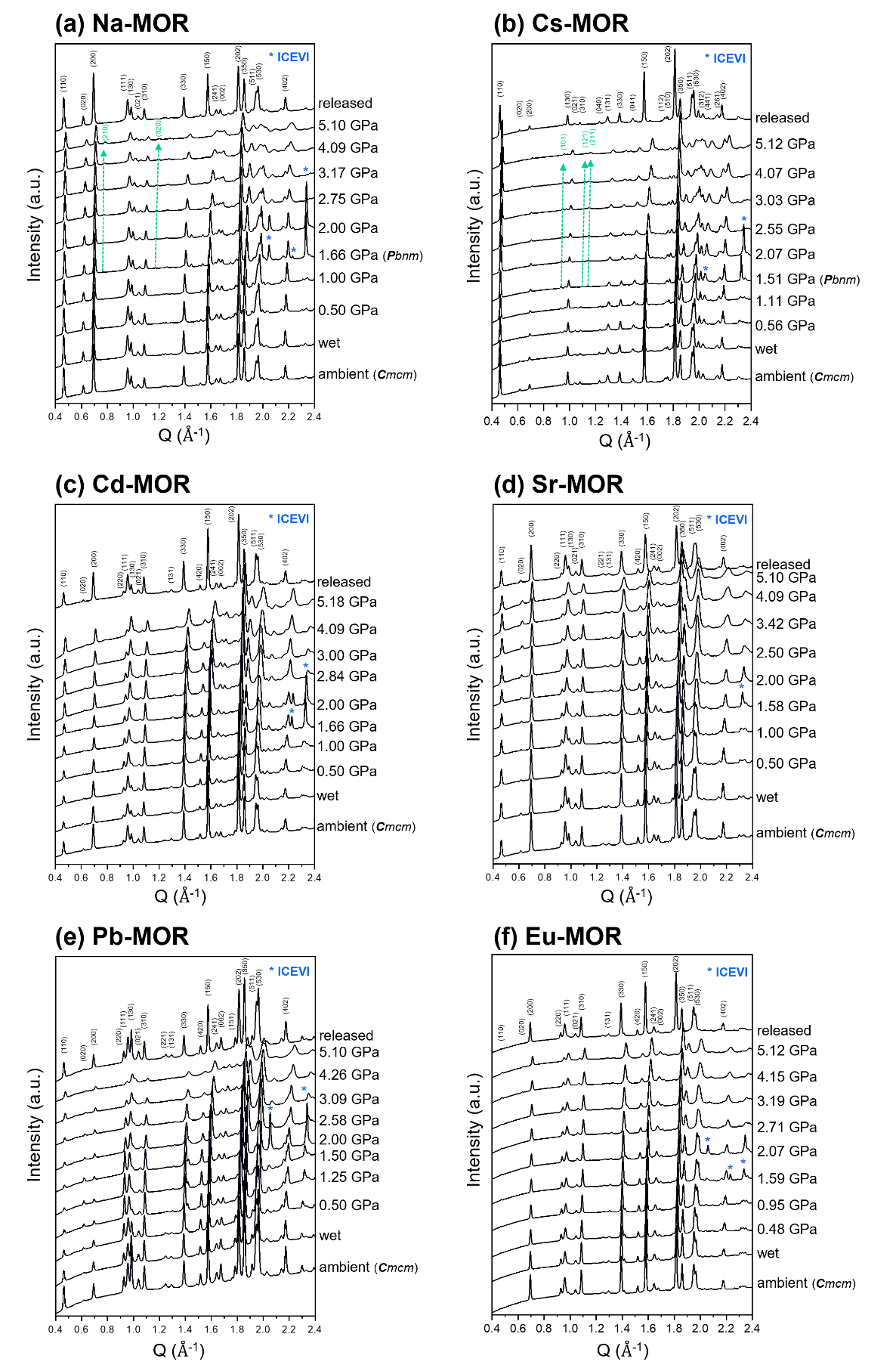


**Figure S2.** Synchrotron X-ray diffraction patterns of ion-exchanged mordenite compressed with water as a PTM.


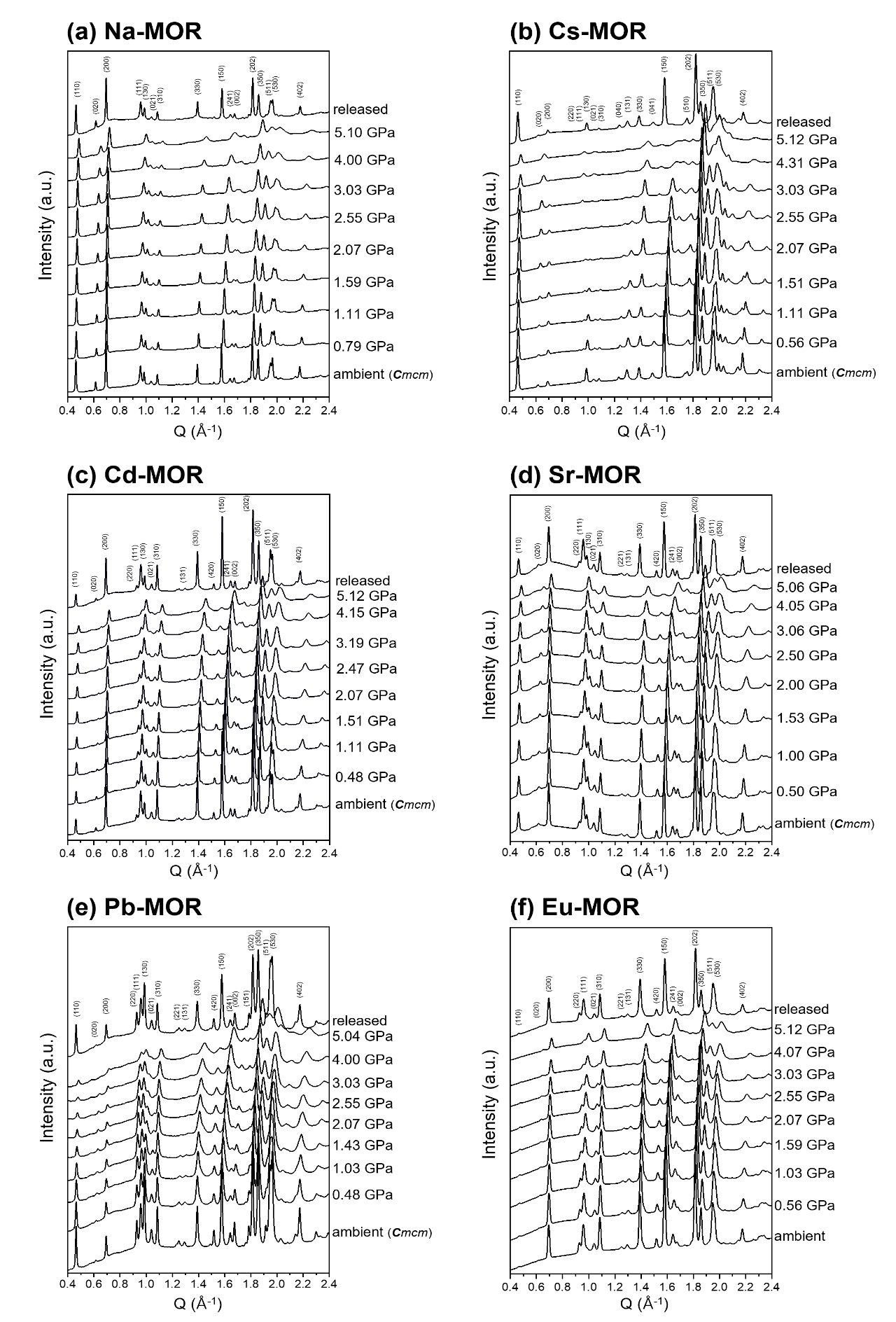
**Figure S3.** Synchrotron X-ray diffraction patterns of ion-exchanged mordenite compressed with s.o. as a PTM.


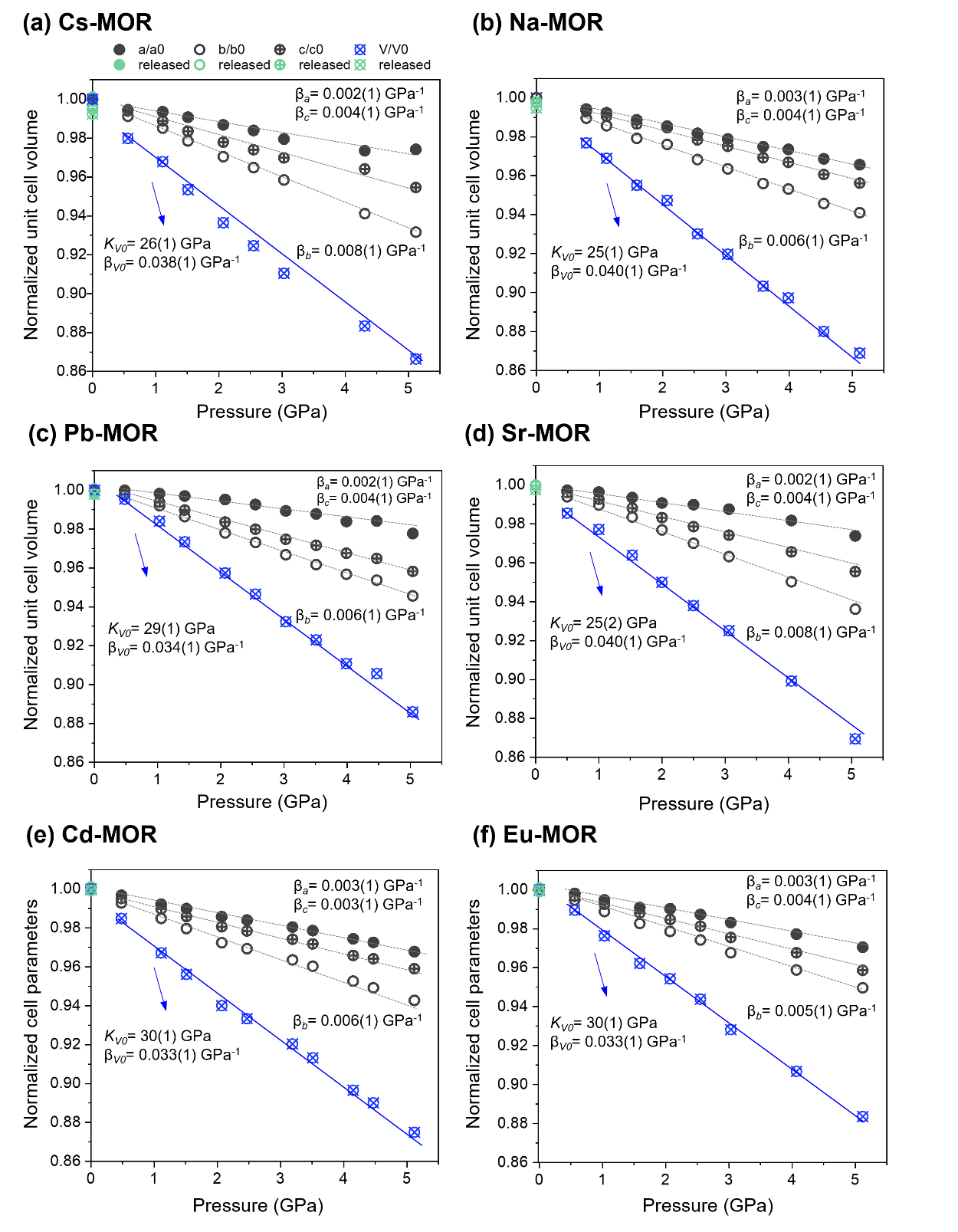


**Figure S4.** Normalized unit-cell volume and parameters of ion-exchanged mordenite (Cs-, Na-, Pb-, Sr-, Cd-, and Eu-MOR) compressed in silicone oil as a function of pressure. The experimental *P-V* data were fitted with a truncated third-order Birch-Murnaghan equation of state using the EoS-Fit7 program. The axial compressibility was calculated using “linearized” equations of state, substituting the cube of a lattice parameter (*a^3^*, *b^3^*, and *c^3^*) for the volume in the equations_._ For *a*, *b*, *c*, and *V*, the esd values are smaller than the size of the symbols.


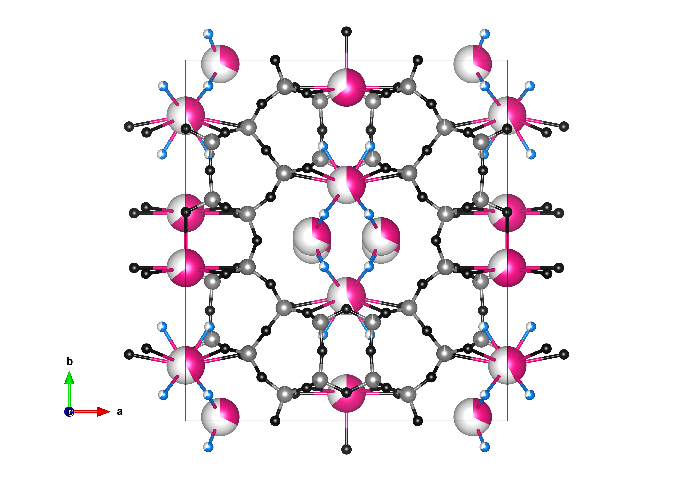

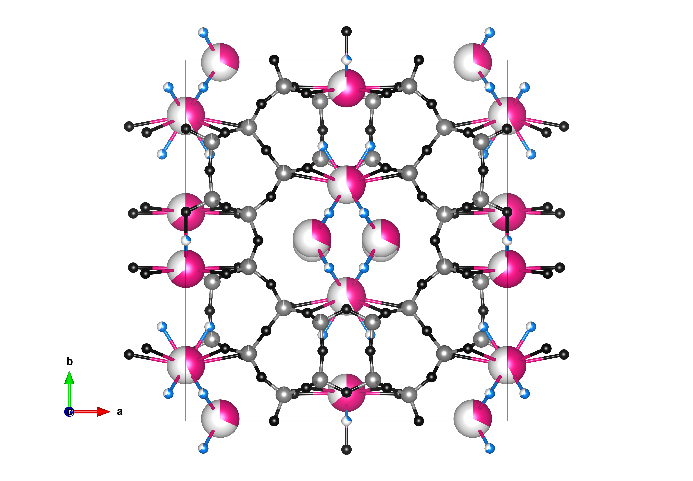


(a) Cs-MOR (ambient) (b) Cs-MOR (wet)


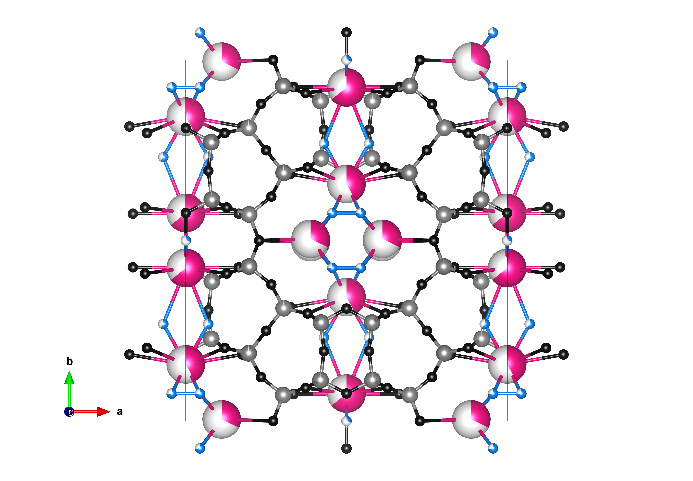

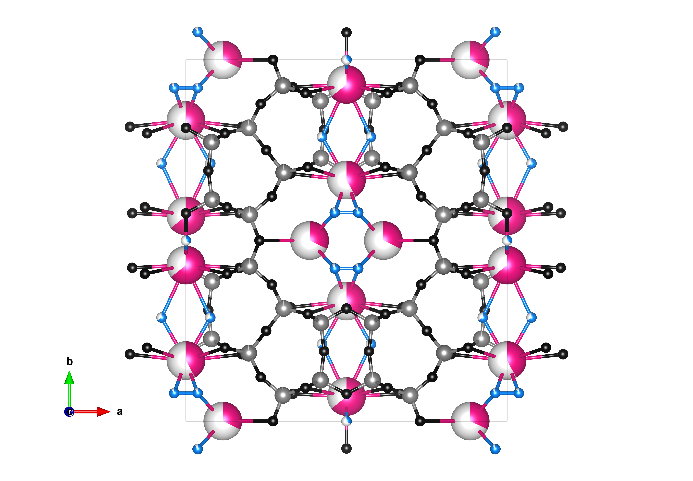


(c) Cs-MOR (0.6(1) GPa) (d) Cs-MOR (1.1(1) GPa)

**Figure S5.** Structural models of cation-exchanged mordenites compressed in water under different pressures. Black and grey circles illustrate framework oxygen atoms and a disordered distribution of Si(Al) atoms, respectively. Magenta, yellow, dark green, light green, orange, red, and blue circles represent Cs^+^, Na^+^, Pb^2+^, Sr^2+^, Cd^2+^, Eu^3+^ ions, and H_2_O molecules, respectively.

**Figure S5.** (continued)


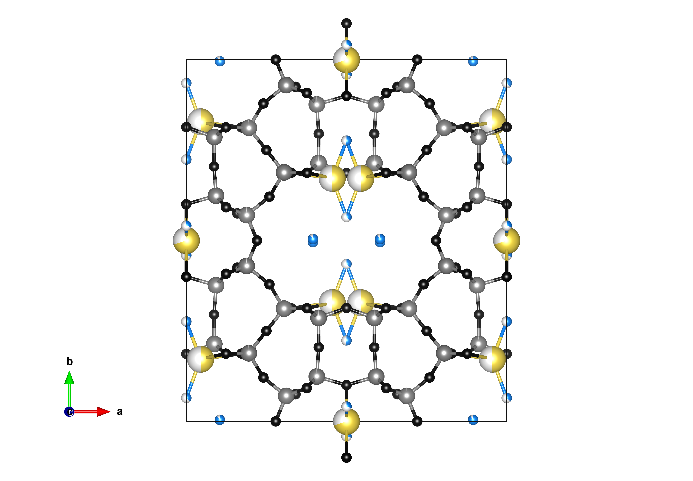

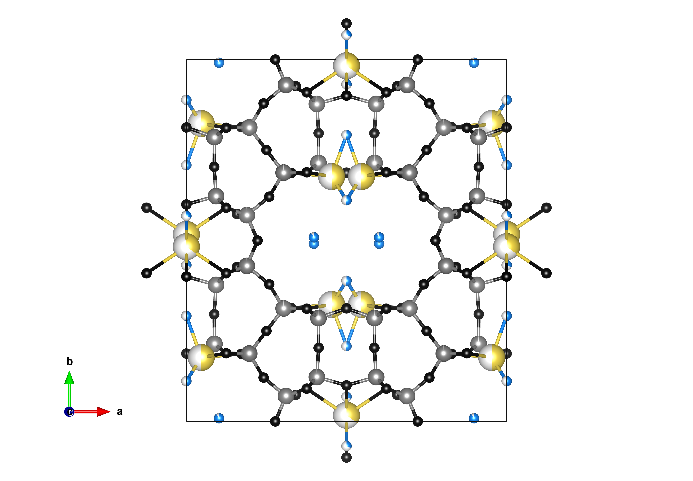


(a) Na-MOR (ambient) (b) Na-MOR (wet)


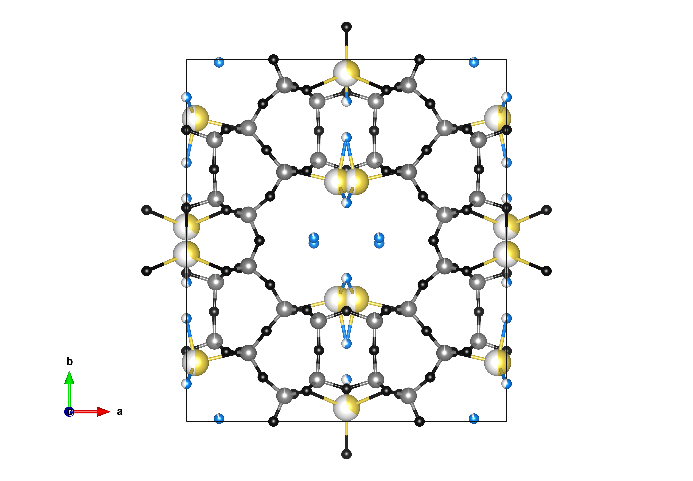

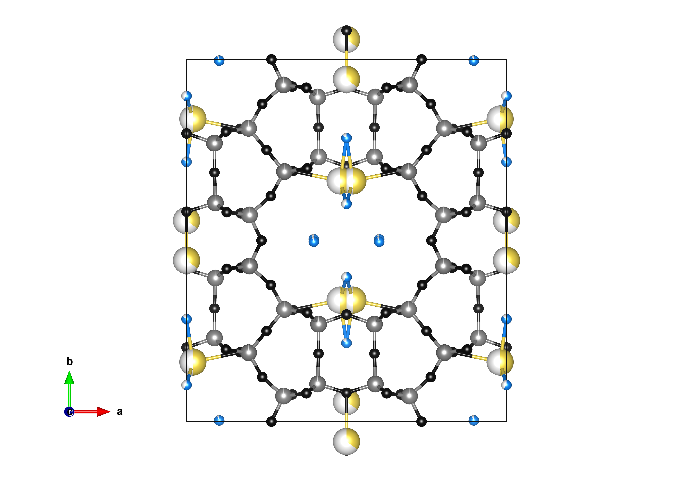


(c) Na-MOR (0.5(1) GPa) (d) Na-MOR (1.0(1) GPa)

**Figure S5.** (continued)


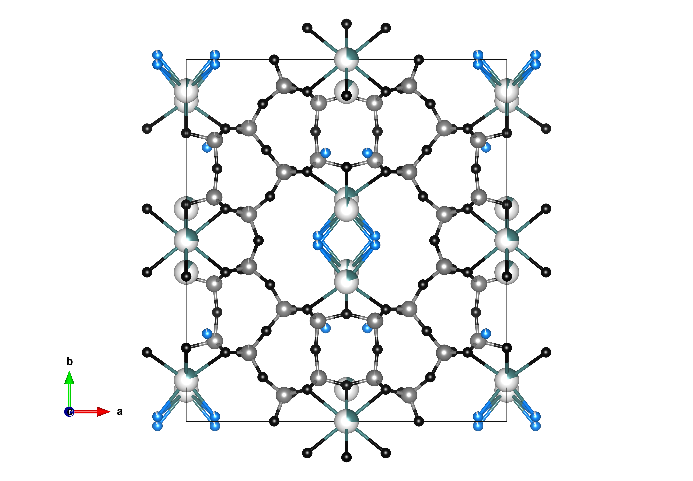

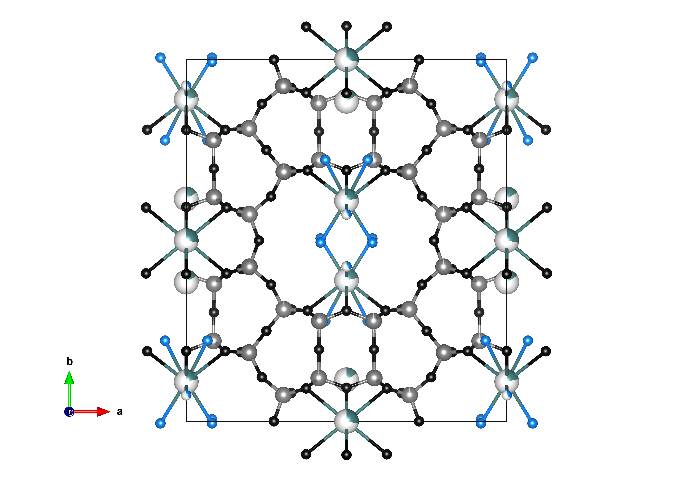


(a) Pb-MOR (ambient) (b) Pb-MOR (wet)


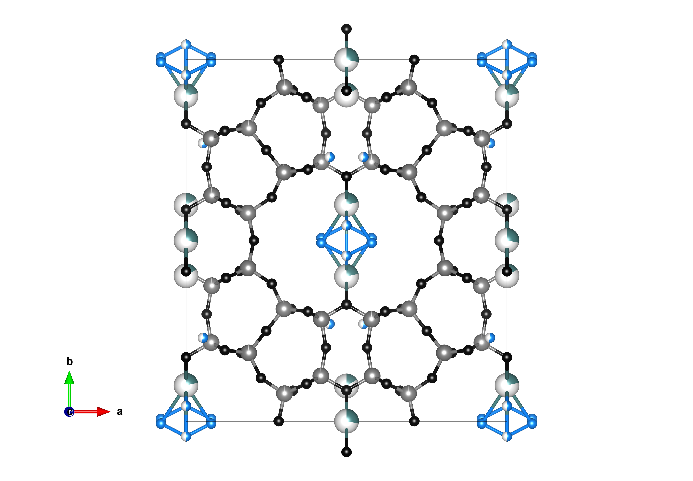

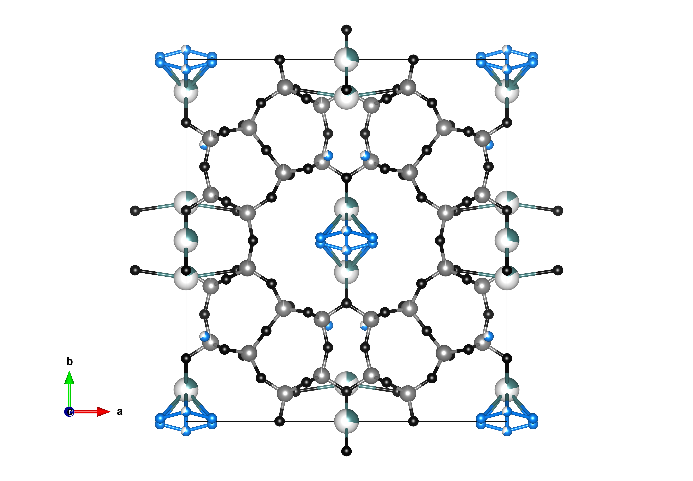


(c) Pb-MOR (0.5(1) GPa) (d) Pb-MOR (1.2(1) GPa)

**Figure S5.** (continued)


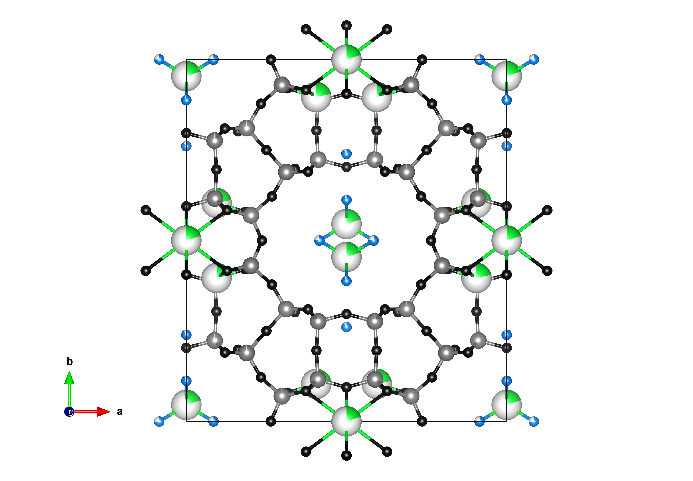

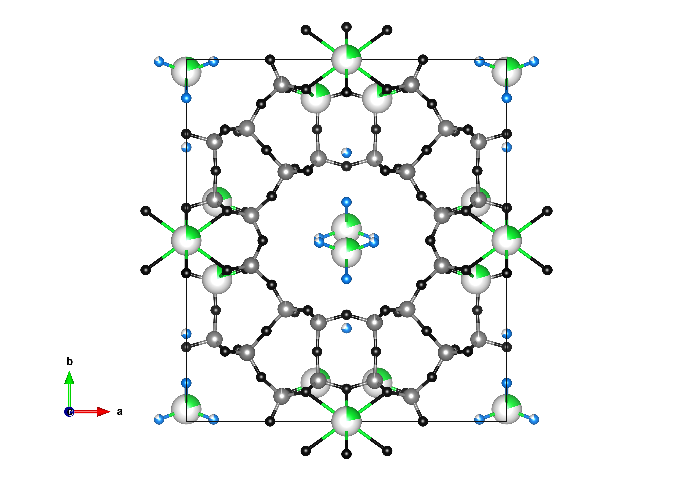


(a) Sr-MOR (ambient) (b) Sr-MOR (wet)


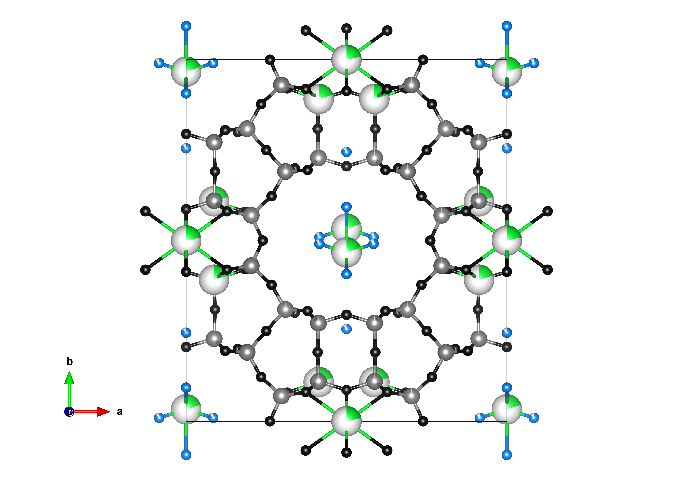

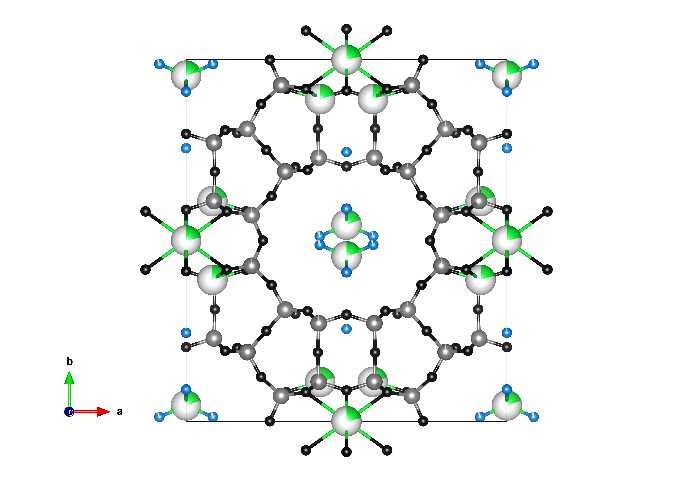


(c) Sr-MOR (0.5(1) GPa) (d) Sr-MOR (1.0(1) GPa)

**Figure S5.** (continued)


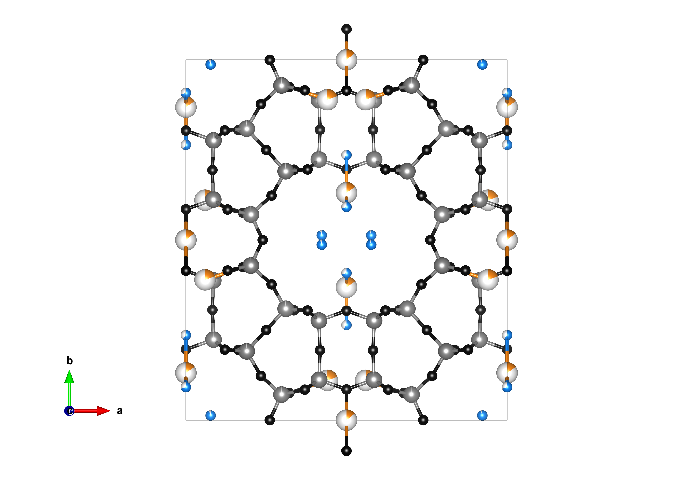

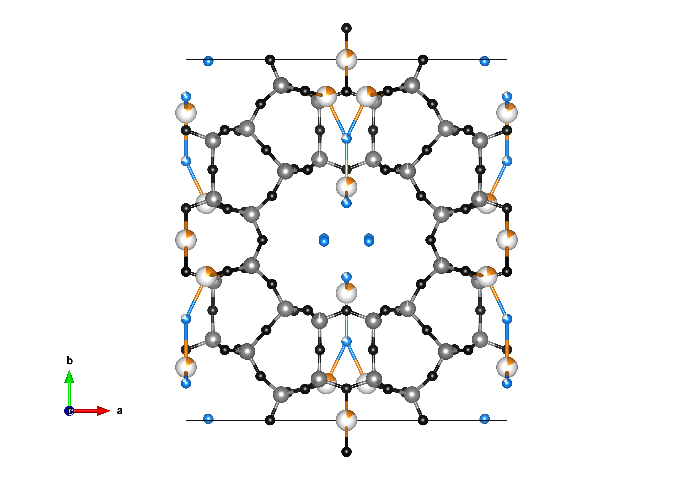


(a) Cd-MOR (ambient ) (b) Cd-MOR (wet)


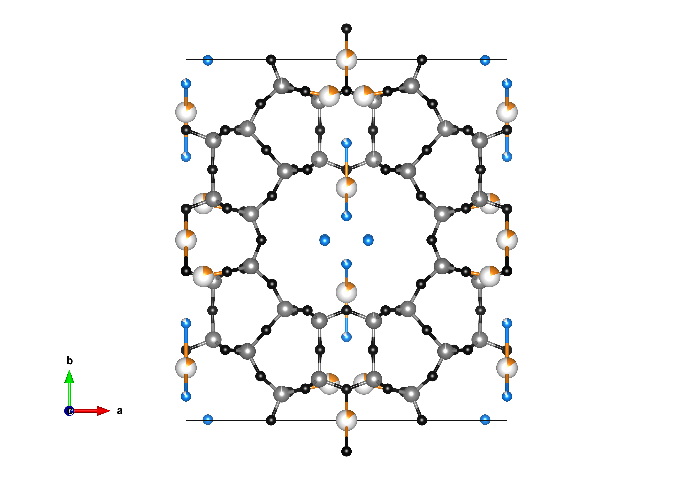

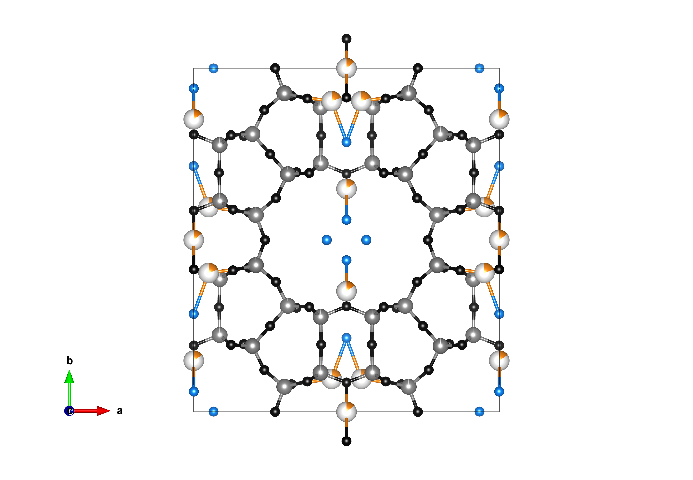


(c) Cd-MOR (0.5(1) GPa) (d) Cd-MOR (1.0(1) GPa)

**Figure S5.** (continued)


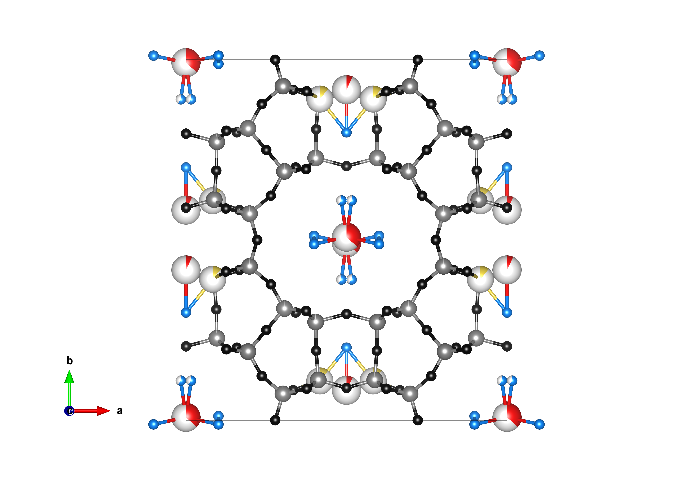

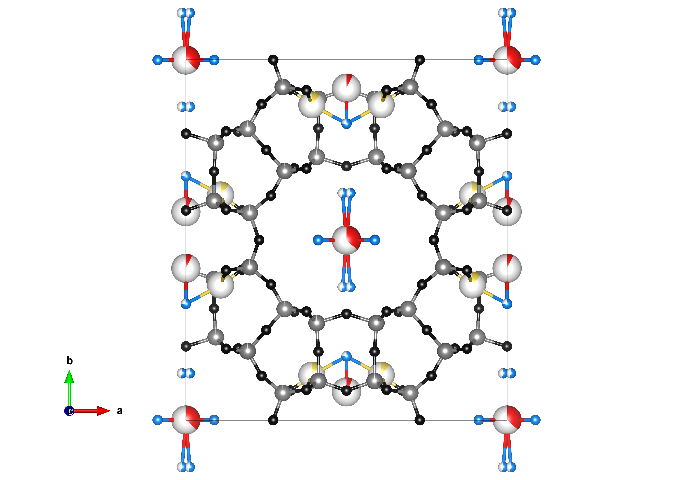


(a) EuNa-MOR (ambient) (b) EuNa-MOR (wet)


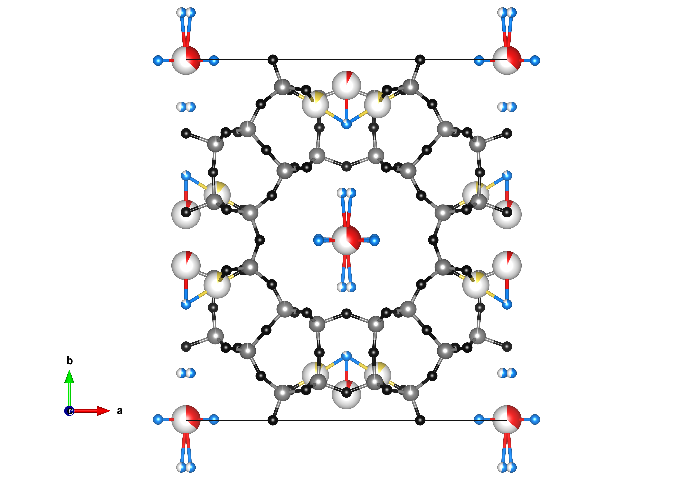

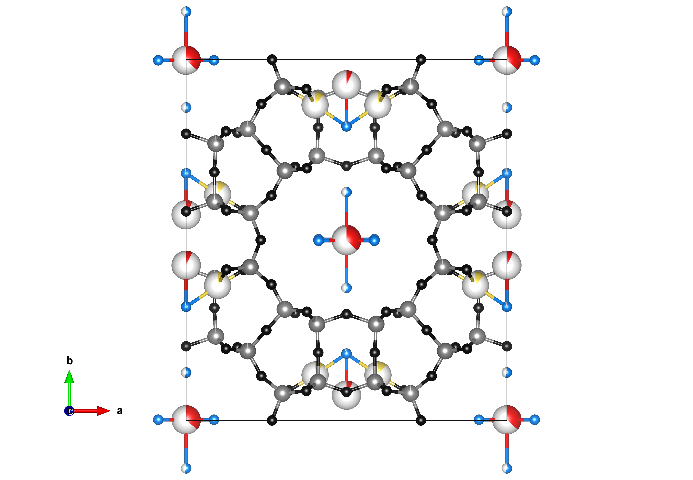


(c) EuNa-MOR (0.5(1) GPa) (d) EuNa-MOR (1.0(1) GPa)

**Table S1.** The results of energy-dispersive X-ray spectroscopy method and thermogravimetric analysis of Eu-mordenite

| Element | Atomic percent (%) | | | | Weight percent of H_2_O (%) | Composition |
| --- | --- | --- | --- | --- | --- | --- |
|  | 1 | 2 | 3 | Average |  |  |
| Si | 26.25 | 25.26 | 24.49 | 25.33 | 14.9(1) | Eu_1.8(1)_Na_1_Al_6.4(4)_Si_41.6(4)_O_96_29.8H_2_O |
| Al | 3.92 | 3.82 | 3.84 | 3.86 |  |  |
| Na | 0.7 | 0.64 | 0.63 | 0.66 |  |  |
| Eu | 1.18 | 1.16 | 1.16 | 1.17 |  |  |

**Table S2.** Refined fractional coordinates for Cs-, Na-, Pb-, Sr-, Cd-, Eu-MOR under different pressures using a H_2_O as a PTM

| Cs-MOR (ambient) | | | | | | |
| --- | --- | --- | --- | --- | --- | --- |
| Refined unit cell composition | | | Cs_6.8_Al_6.8_Si_41.2_O_96_ ·19.6H_2_O (_w_*R*_p_ 2.246%, χ^2^ 1.08) | | | |
| Refined unit cell parameters | | | *a* 18.1763(15) Å, *b* 20.4057(19) Å, *c* 7.4928(6) Å, *V* 2779.1(7) Å^3^ | | | |
| Atom, site | Occupancy | x^a^ | | y^a^ | z^a^ | *U_iso_*^a,b^ |
| Si(1), 16a | 0.9899 | 0.1918(4) | | 0.4264(3) | 0.5443(9) | 0.0531(20) |
| Si(2), 16a | 0.9899 | 0.1948(4) | | 0.1866(4) | 0.5384(8) | 0.0531(20) |
| Si(3), 8b | 0.9899 | 0.0827(4) | | 0.3866(5) | 0.25000 | 0.0531(20) |
| Si(4), 8b | 0.9899 | 0.0811(5) | | 0.2264(4) | 0.25000 | 0.0531(20) |
| O(1), 16a | 1.0000 | 0.1242(4) | | 0.4082(5) | 0.4283(6) | 0.077(4) |
| O(2), 16a | 1.0000 | 0.1219(4) | | 0.2004(5) | 0.4297(6) | 0.077(4) |
| O(3), 16a | 1.0000 | 0.2640(5) | | 0.3789(4) | 0.5251(16) | 0.077(4) |
| O(4), 8b | 1.0000 | 0.0753(8) | | 0.3057(4) | 0.25000 | 0.077(4) |
| O(5), 8b | 1.0000 | 0.1762(7) | | 0.8157(6) | 0.25000 | 0.077(4) |
| O(6), 8b | 1.0000 | 0.1607(7) | | 0.5760(6) | 0.25000 | 0.077(4) |
| O(7), 8b | 1.0000 | 0.2219(7) | | 0.50000 | 0.50000 | 0.077(4) |
| O(8), 8b | 1.0000 | 0.25000 | | 0.25000 | 0.50000 | 0.077(4) |
| O(9), 4c | 1.0000 | 0.00000 | | 0.4213(7) | 0.25000 | 0.077(4) |
| O(10), 4c | 1.0000 | 0.00000 | | 0.1900(8) | 0.25000 | 0.077(4) |
| Cs(1), 4c | 0.6401 | 0.00000 | | 0.5761(5) | 0.25000 | 0.261(5) |
| Cs(2), 4c | 0.4211 | 0.00000 | | 0.8459(7) | 0.25000 | 0.261(5) |
| Cs(3), 8b | 0.3229 | 0.1075(7) | | -0.0112(6) | 0.25000 | 0.261(5) |
| OW(1), 16a | 0.5750 | -0.0736(16) | | 0.7389(10) | 0.29(4) | 0.261(5) |
| OW(2), 16a | 0.6500 | 0.0700(13) | | 0.9278(8) | 0.5384(26) | 0.261(5) |

| Cs-MOR (wet) | | | | | | | |
| --- | --- | --- | --- | --- | --- | --- | --- |
| Refined unit cell composition | | | | Cs_6.8_Al_6.8_Si_41.2_O_96_ ·21.5H_2_O (_w_*R*_p_ 1.479%, χ^2^ 0.72) | | | |
| Refined unit cell parameters | | | | *a* 18.1824(13) Å, *b* 20.4307(17) Å, *c* 7.4977(6) Å, *V* 2785.2(6) Å^3^ | | | |
| Atom, site | Occupancy | x^a^ | | | y^a^ | z^a^ | *U_iso_*^a,b^ |
| Si(1), 16a | 0.9899 | 0.1949(4) | | | 0.42730(29) | 0.5424(8) | 0.0516(19) |
| Si(2), 16a | 0.9899 | 0.1956(4) | | | 0.1867(3) | 0.5378(7) | 0.0516(19) |
| Si(3), 8b | 0.9899 | 0.0822(4) | | | 0.3861(4) | 0.25000 | 0.0516(19) |
| Si(4), 8b | 0.9899 | 0.0806(4) | | | 0.2263(4) | 0.25000 | 0.0516(19) |
| O(1), 16a | 1.0000 | 0.1258(4) | | | 0.4082(4) | 0.4271(5) | 0.070(4) |
| O(2), 16a | 1.0000 | 0.12189(30) | | | 0.2007(4) | 0.4293(5) | 0.070(4) |
| O(3), 16a | 1.0000 | 0.2647(4) | | | 0.37864(29) | 0.5257(15) | 0.070(4) |
| O(4), 8b | 1.0000 | 0.0742(7) | | | 0.3057(4) | 0.25000 | 0.070(4) |
| O(5), 8b | 1.0000 | 0.1767(6) | | | 0.8158(6) | 0.25000 | 0.070(4) |
| O(6), 8b | 1.0000 | 0.1636(7) | | | 0.5751(6) | 0.25000 | 0.070(4) |
| O(7), 8b | 1.0000 | 0.2252(7) | | | 0.50000 | 0.50000 | 0.070(4) |
| O(8), 8b | 1.0000 | 0.25000 | | | 0.25000 | 0.50000 | 0.070(4) |
| O(9), 4c | 1.0000 | 0.00000 | | | 0.4207(6) | 0.25000 | 0.070(4) |
| O(10), 4c | 1.0000 | 0.00000 | | | 0.1902(7) | 0.25000 | 0.070(4) |
| Cs(1), 4c | 0.6401 | 0.00000 | | | 0.5782(5) | 0.25000 | 0.257(22) |
| Cs(2), 4c | 0.4211 | 0.00000 | | | 0.8453(8) | 0.25000 | 0.257(22) |
| Cs(3), 8b | 0.3229 | 0.1072(7) | | | -0.0065(9) | 0.25000 | 0.257(22) |
| OW(1), 16a | 0.607(17) | -0.0741(19) | | | 0.7388(11) | 0.225(29) | 0.257(22) |
| OW(2), 16a | 0.669(19) | 0.0544(18) | | | 0.9241(10) | 0.514(5) | 0.257(22) |
| OW(3), 4c | 0.26(3) | 0.00000 | | | 0.50000 | 0.00000 | 0.257(22) |
| Cs-MOR (0.6(1) GPa) | | | | | | | |
| Refined unit cell composition | | | Cs_6.8_Al_6.8_Si_41.2_O_96_ ·22.5H_2_O (_w_*R*_p_ 1.631%, χ^2^ 0.65) | | | | |
| Refined unit cell parameters | | | *a* 18.1217(14) Å, *b* 20.3583(18), *c* 7.4692(6), *V* 2755.6(7) Å^3^ | | | | |
| Atom, site | Occupancy | x^a^ | | | y^a^ | z^a^ | *U_iso_*^a,b^ |
| Si(1), 16a | 0.9899 | 0.1970(4) | | | 0.42795(29) | 0.5419(8) | 0.0518(20) |
| Si(2), 16a | 0.9899 | 0.1954(4) | | | 0.1876(3) | 0.5383(7) | 0.0518(20) |
| Si(3), 8b | 0.9899 | 0.0810(4) | | | 0.3868(4) | 0.25000 | 0.0518(20) |
| Si(4), 8b | 0.9899 | 0.0791(4) | | | 0.2274(4) | 0.25000 | 0.0518(20) |
| O(1), 16a | 1.0000 | 0.1272(3) | | | 0.4084(5) | 0.4264(5) | 0.077(4) |
| O(2), 16a | 1.0000 | 0.12093(29) | | | 0.2024(4) | 0.4298(5) | 0.077(4) |
| O(3), 16a | 1.0000 | 0.2660(4) | | | 0.37829(28) | 0.5280(14) | 0.077(4) |
| O(4), 8b | 1.0000 | 0.0701(7) | | | 0.3066(4) | 0.25000 | 0.077(4) |
| O(5), 8b | 1.0000 | 0.1754(6) | | | 0.8157(6) | 0.25000 | 0.077(4) |
| O(6), 8b | 1.0000 | 0.1648(6) | | | 0.5743(6) | 0.25000 | 0.077(4) |
| O(7), 8b | 1.0000 | 0.2283(7) | | | 0.50000 | 0.50000 | 0.077(4) |
| O(8), 8b | 1.0000 | 0.25000 | | | 0.25000 | 0.50000 | 0.077(4) |
| O(9), 4c | 1.0000 | 0.00000 | | | 0.4238(6) | 0.25000 | 0.077(4) |
| O(10), 4c | 1.0000 | 0.00000 | | | 0.1886(6) | 0.25000 | 0.077(4) |
| Cs(1), 4c | 0.6401 | 0.00000 | | | 0.5760(5) | 0.25000 | 0.256(20) |
| Cs(2), 4c | 0.4211 | 0.00000 | | | 0.8432(8) | 0.25000 | 0.256(20) |
| Cs(3), 8b | 0.3229 | 0.1118(7) | | | -0.0045(8) | 0.25000 | 0.256(20) |
| OW(1), 16a | 0.599(17) | -0.0699(17) | | | 0.7312(12) | 0.2(6) | 0.256(20) |
| OW(2), 16a | 0.738(18) | 0.0439(19) | | | 0.9241(9) | 0.505(4) | 0.256(20) |
| OW(3), 4c | 0.29(3) | 0.00000 | | | 0.50000 | 0.00000 | 0.256(20) |

| Cs-MOR (1.1(1) GPa) | | | | | | |
| --- | --- | --- | --- | --- | --- | --- |
| Refined unit cell composition | | | Cs_6.8_Al_6.8_Si_41.2_O_96_ ·26.4H_2_O (_w_*R*_p_ 1.57%, χ^2^ 0.74) | | | |
| Refined unit cell parameters | | | *a* 18.0604(14) Å, *b* 20.3196(19), *c* 7.4420(6), *V* 2731.1(7) Å^3^ | | | |
| Atom, site | Occupancy | x^a^ | | y^a^ | z^a^ | *U_iso_*^a,b^ |
| Si(1), 16a | 0.9899 | 0.1981(4) | | 0.42865(31) | 0.5398(8) | 0.0545(21) |
| Si(2), 16a | 0.9899 | 0.1955(4) | | 0.1881(3) | 0.5395(8) | 0.0545(21) |
| Si(3), 8b | 0.9899 | 0.0812(4) | | 0.3867(5) | 0.25000 | 0.0545(21) |
| Si(4), 8b | 0.9899 | 0.0795(4) | | 0.2278(4) | 0.25000 | 0.0545(21) |
| O(1), 16a | 1.0000 | 0.1279(4) | | 0.4079(5) | 0.4268(5) | 0.086(4) |
| O(2), 16a | 1.0000 | 0.12096(31) | | 0.2032(4) | 0.4302(5) | 0.086(4) |
| O(3), 16a | 1.0000 | 0.2673(4) | | 0.37815(30) | 0.5263(16) | 0.086(4) |
| O(4), 8b | 1.0000 | 0.0698(7) | | 0.3067(4) | 0.25000 | 0.086(4) |
| O(5), 8b | 1.0000 | 0.1744(6) | | 0.8148(6) | 0.25000 | 0.086(4) |
| O(6), 8b | 1.0000 | 0.1670(7) | | 0.5755(6) | 0.25000 | 0.086(4) |
| O(7), 8b | 1.0000 | 0.2290(7) | | 0.50000 | 0.50000 | 0.086(4) |
| O(8), 8b | 1.0000 | 0.25000 | | 0.25000 | 0.50000 | 0.086(4) |
| O(9), 4c | 1.0000 | 0.00000 | | 0.4245(6) | 0.25000 | 0.086(4) |
| O(10), 4c | 1.0000 | 0.00000 | | 0.1883(7) | 0.25000 | 0.086(4) |
| Cs(1), 4c | 0.6401 | 0.00000 | | 0.5681(6) | 0.25000 | 0.280(17) |
| Cs(2), 4c | 0.4211 | 0.00000 | | 0.8323(8) | 0.25000 | 0.280(17) |
| Cs(3), 8b | 0.3229 | 0.1149(7) | | -0.0002(7) | 0.25000 | 0.280(17) |
| OW(1), 16a | 0.656(17) | -0.0759(14) | | 0.7134(12) | 3.25(3) | 0.280(17) |
| OW(2), 16a | 0.902(18) | 0.0366(19) | | 0.9225(9) | 0.4866(30) | 0.280(17) |
| OW(3), 4c | 0.35(4) | 0.00000 | | 0.50000 | 0.00000 | 0.280(17) |

| Na-MOR (ambient) | | | | | | |
| --- | --- | --- | --- | --- | --- | --- |
| Refined unit cell composition | | | Na_6.6(2)_Al_6.6(2)_Si_41.4(2)_O_96_ ·21.4H_2_O (_w_*R*_p_ 2.565%, χ^2^ 1.64) | | | |
| Refined unit cell parameters | | | *a* 18.0880(21) Å, *b* 20.4247(22) Å, *c* 7.5076(8) Å, *V* 2773.6(8) Å^3^ | | | |
| Atom, site | Occupancy | x^a^ | | y^a^ | z^a^ | *U_iso_*^a,b^ |
| Si(1), 16a | 0.9902 | 0.1884(6) | | 0.4297(4) | 0.5410(11) | 0.0253(23) |
| Si(2), 16a | 0.9902 | 0.1951(5) | | 0.1883(5) | 0.5355(11) | 0.0253(23) |
| Si(3), 8b | 0.9902 | 0.0927(6) | | 0.3761(6) | 0.25000 | 0.0253(23) |
| Si(4), 8b | 0.9902 | 0.0872(6) | | 0.2140(7) | 0.25000 | 0.0253(23) |
| O(1), 16a | 1.0000 | 0.1241(6) | | 0.4081(7) | 0.4278(7) | 0.054(5) |
| O(2), 16a | 1.0000 | 0.1233(5) | | 0.1872(7) | 0.4303(8) | 0.054(5) |
| O(3), 16a | 1.0000 | 0.2594(6) | | 0.3789(4) | 0.5140(25) | 0.054(5) |
| O(4), 8b | 1.0000 | 0.0867(13) | | 0.2954(6) | 0.25000 | 0.054(5) |
| O(5), 8b | 1.0000 | 0.1726(10) | | 0.1873(10) | 0.75000 | 0.054(5) |
| O(6), 8b | 1.0000 | 0.1592(11) | | 0.4250(9) | 0.75000 | 0.054(5) |
| O(7), 8b | 1.0000 | 0.2210(10) | | 0.50000 | 0.50000 | 0.054(5) |
| O(8), 8b | 1.0000 | 0.25000 | | 0.25000 | 0.50000 | 0.054(5) |
| O(9), 4c | 1.0000 | 0.00000 | | 0.3998(11) | 0.25000 | 0.054(5) |
| O(10), 4c | 1.0000 | 0.00000 | | 0.1864(11) | 0.25000 | 0.054(5) |
| Na(1), 4c | 0.7000 | 0.00000 | | 0.50000 | 0.00000 | 0.056(10) |
| Na(2), 8b | 0.5000 | 0.4554(21) | | 0.3280(24) | 0.25000 | 0.056(10) |
| OW(1), 4c | 0.3000 | 0.00000 | | 0.459(7) | 0.75000 | 0.056(10) |
| OW(2), 8b | 0.5349 | 0.00000 | | 0.7240(23) | 0.311(8) | 0.056(10) |
| OW(3), 16a | 0.9000 | -0.3953(10) | | 0.5042(14) | -0.166(3) | 0.056(10) |
| OW(4), 4c | 0.3728 | 0.00000 | | 1.0649(25) | 0.75000 | 0.056(10) |

| Na-MOR (wet) | | | | | | |
| --- | --- | --- | --- | --- | --- | --- |
| Refined unit cell composition | | | Na_6.6(2)_Al_6.6(2)_Si_41.4(2)_O_96_ ·24.2H_2_O (_w_*R*_p_ 2.629%, χ^2^ 1.72) | | | |
| Refined unit cell parameters | | | *a* 18.0993(19) Å, *b* 20.4542(21) Å, *c* 7.5102(7) Å, *V* 2780.3(8) Å^3^ | | | |
| Atom, site | Occupancy | x^a^ | | y^a^ | z^a^ | *U_iso_*^a,b^ |
| Si(1), 16a | 0.9902 | 0.1889(6) | | 0.4299(4) | 0.5377(11) | 0.0313(22) |
| Si(2), 16a | 0.9902 | 0.1962(5) | | 0.1876(5) | 0.5337(10) | 0.0313(22) |
| Si(3), 8b | 0.9902 | 0.0926(6) | | 0.3773(6) | 0.25000 | 0.0313(22) |
| Si(4), 8b | 0.9902 | 0.0876(6) | | 0.2144(7) | 0.25000 | 0.0313(22) |
| O(1), 16a | 1.0000 | 0.1244(6) | | 0.4094(6) | 0.4277(8) | 0.050(4) |
| O(2), 16a | 1.0000 | 0.1243(5) | | 0.1884(7) | 0.4304(8) | 0.050(4) |
| O(3), 16a | 1.0000 | 0.2588(6) | | 0.3789(4) | 0.5154(24) | 0.050(4) |
| O(4), 8b | 1.0000 | 0.0870(12) | | 0.2964(6) | 0.25000 | 0.050(4) |
| O(5), 8b | 1.0000 | 0.1718(9) | | 0.1868(10) | 0.75000 | 0.050(4) |
| O(6), 8b | 1.0000 | 0.1580(10) | | 0.4265(9) | 0.75000 | 0.050(4) |
| O(7), 8b | 1.0000 | 0.2224(9) | | 0.50000 | 0.50000 | 0.050(4) |
| O(8), 8b | 1.0000 | 0.25000 | | 0.25000 | 0.50000 | 0.050(4) |
| O(9), 4c | 1.0000 | 0.00000 | | 0.4004(12) | 0.25000 | 0.050(4) |
| O(10), 4c | 1.0000 | 0.00000 | | 0.1875(11) | 0.25000 | 0.050(4) |
| Na(1), 4c | 0.3500 | 0.00000 | | 0.483(7) | -0.046(12) | 0.035(8) |
| Na(2), 8b | 0.5000 | 0.4534(18) | | 0.3232(22) | 0.25000 | 0.035(8) |
| OW(1), 4c | 0.3226 | 0.00000 | | 0.432(7) | 0.75000 | 0.035(8) |
| OW(2), 8b | 0.5349 | 0.00000 | | 0.7082(29) | 0.199(6) | 0.035(8) |
| OW(3), 16a | 0.907(11) | -0.3981(9) | | 0.5088(12) | -0.1625(26) | 0.035(8) |
| OW(4), 8b | 0.5157 | 0.00000 | | 1.1114(30) | -0.060(8) | 0.035(8) |

| Na-MOR (0.5(1) GPa) | | | | | | |
| --- | --- | --- | --- | --- | --- | --- |
| Refined unit cell composition | | | Na_6.6(2)_Al_6.6(2)_Si_41.4(2)_O_96_ ·25.1H_2_O (_w_*R*_p_ 2.714%, χ^2^ 1.89) | | | |
| Refined unit cell parameters | | | *a* 18.0441(23) Å, *b* 20.4034(25) Å, *c* 7.4871(9) Å, *V* 2756.5(9) Å^3^ | | | |
| Atom, site | Occupancy | x^a^ | | y^a^ | z^a^ | *U_iso_*^a,b^ |
| Si(1), 16a | 0.9902 | 0.1935(6) | | 0.4292(5) | 0.5387(12) | 0.046(3) |
| Si(2), 16a | 0.9902 | 0.1936(6) | | 0.1891(6) | 0.5342(12) | 0.046(3) |
| Si(3), 8b | 0.9902 | 0.0917(6) | | 0.3851(8) | 0.25000 | 0.046(3) |
| Si(4), 8b | 0.9902 | 0.0878(7) | | 0.2217(8) | 0.25000 | 0.046(3) |
| O(1), 16a | 1.0000 | 0.1247(6) | | 0.4156(8) | 0.4292(8) | 0.069(6) |
| O(2), 16a | 1.0000 | 0.1237(5) | | 0.1924(8) | 0.4290(8) | 0.069(6) |
| O(3), 16a | 1.0000 | 0.2616(7) | | 0.3780(5) | 0.5054(28) | 0.069(6) |
| O(4), 8b | 1.0000 | 0.0864(15) | | 0.3033(7) | 0.25000 | 0.069(6) |
| O(5), 8b | 1.0000 | 0.1675(10) | | 0.1916(11) | 0.75000 | 0.069(6) |
| O(6), 8b | 1.0000 | 0.1653(12) | | 0.4240(10) | 0.75000 | 0.069(6) |
| O(7), 8b | 1.0000 | 0.2282(11) | | 0.50000 | 0.50000 | 0.069(6) |
| O(8), 8b | 1.0000 | 0.25000 | | 0.25000 | 0.50000 | 0.069(6) |
| O(9), 4c | 1.0000 | 0.00000 | | 0.4097(14) | 0.25000 | 0.069(6) |
| O(10), 4c | 1.0000 | 0.00000 | | 0.1945(14) | 0.25000 | 0.069(6) |
| Na(1), 4c | 0.3500 | 0.00000 | | 0.462(4) | -0.120(8) | 0.030(12) |
| Na(2), 8b | 0.5000 | 0.472(3) | | 0.3372(19) | 0.25000 | 0.030(12) |
| OW(1), 4c | 0.3753 | 0.00000 | | 0.384(8) | 0.75000 | 0.030(12) |
| OW(2), 8b | 0.5782 | 0.00000 | | 0.7151(27) | 0.196(9) | 0.030(12) |
| OW(3), 16a | 0.9104 | -0.3977(11) | | 0.5078(14) | -0.164(3) | 0.030(12) |
| OW(4), 8b | 0.5494 | 0.00000 | | 1.1041(28) | 0.010(8) | 0.030(12) |

| Na-MOR (1.0(1) GPa) | | | | | | |
| --- | --- | --- | --- | --- | --- | --- |
| Refined unit cell composition | | | Na_6.6(2)_Al_6.6(2)_Si_41.4(2)_O_96_ ·26.4H_2_O (_w_*R*_p_ 2.752%, χ^2^ 1.95) | | | |
| Refined unit cell parameters | | | *a* 17.9725(24) Å, *b* 20.3503(26) Å, *c* 7.4589(9) Å, *V* 2728.1(10) Å^3^ | | | |
| Atom, site | Occupancy | x^a^ | | y^a^ | z^a^ | *U_iso_*^a,b^ |
| Si(1), 16a | 0.9902 | 0.1968(6) | | 0.4295(5) | 0.5375(13) | 0.043(3) |
| Si(2), 16a | 0.9902 | 0.1939(5) | | 0.1902(5) | 0.5328(13) | 0.043(3) |
| Si(3), 8b | 0.9902 | 0.0902(6) | | 0.3963(7) | 0.25000 | 0.043(3) |
| Si(4), 8b | 0.9902 | 0.0872(7) | | 0.2312(7) | 0.25000 | 0.043(3) |
| O(1), 16a | 1.0000 | 0.1254(6) | | 0.4218(7) | 0.4308(8) | 0.079(6) |
| O(2), 16a | 1.0000 | 0.1226(5) | | 0.1992(7) | 0.4292(8) | 0.079(6) |
| O(3), 16a | 1.0000 | 0.2634(7) | | 0.3771(5) | 0.5053(28) | 0.079(6) |
| O(4), 8b | 1.0000 | 0.0873(15) | | 0.3123(6) | 0.25000 | 0.079(6) |
| O(5), 8b | 1.0000 | 0.1680(10) | | 0.1916(11) | 0.75000 | 0.079(6) |
| O(6), 8b | 1.0000 | 0.1696(11) | | 0.4250(10) | 0.75000 | 0.079(6) |
| O(7), 8b | 1.0000 | 0.2346(10) | | 0.50000 | 0.50000 | 0.079(6) |
| O(8), 8b | 1.0000 | 0.25000 | | 0.25000 | 0.50000 | 0.079(6) |
| O(9), 4c | 1.0000 | 0.00000 | | 0.4205(13) | 0.25000 | 0.079(6) |
| O(10), 4c | 1.0000 | 0.00000 | | 0.2043(14) | 0.25000 | 0.079(6) |
| Na(1), 4c | 0.3500 | 0.00000 | | 0.445(3) | -0.192(11) | 0.042(24) |
| Na(2), 8b | 0.5000 | 0.480(4) | | 0.3355(23) | 0.25000 | 0.042(24) |
| OW(2), 8b | 0.8478 | 0.00000 | | 0.7170(19) | 0.166(4) | 0.042(24) |
| OW(3), 16a | 0.9254 | -0.3978(10) | | 0.4967(13) | -0.164(4) | 0.042(24) |
| OW(4), 8b | 0.6043 | 0.00000 | | 1.1009(29) | 0.019(8) | 0.042(24) |

| Pb-MOR (ambient) | | | | | | |
| --- | --- | --- | --- | --- | --- | --- |
| Refined unit cell composition | | | Pb_3.2(2)_Al_6.3(5)_Si_41.6(4)_O_96_ ·22.4H_2_O (_w_*R*_p_ 2.617%, χ^2^ 1.00) | | | |
| Refined unit cell parameters | | | *a* 18.0951(30) Å, *b* 20.413(4) Å, *c* 7.4995(13) Å, *V* 2770.2(13) Å^3^ | | | |
| Atom, site | Occupancy | x^a^ | | y^a^ | z^a^ | *U_iso_*^a,b^ |
| Si(1), 16a | 0.9906 | 0.1950(5) | | 0.4278(4) | 0.5411(10) | 0.046(4) |
| Si(2), 16a | 0.9906 | 0.1952(4) | | 0.1893(4) | 0.5381(10) | 0.046(4) |
| Si(3), 8b | 0.9906 | 0.0871(5) | | 0.3804(7) | 0.25000 | 0.046(4) |
| Si(4), 8b | 0.9906 | 0.0892(5) | | 0.2222(7) | 0.25000 | 0.046(4) |
| O(1), 16a | 1.0000 | 0.1224(5) | | 0.4121(6) | 0.4289(6) | 0.050(5) |
| O(2), 16a | 1.0000 | 0.1224(4) | | 0.1906(6) | 0.4281(6) | 0.050(5) |
| O(3), 16a | 1.0000 | 0.2617(5) | | 0.3780(3) | 0.4975(22) | 0.050(5) |
| O(4), 8b | 1.0000 | 0.0966(13) | | 0.3016(6) | 0.25000 | 0.050(5) |
| O(5), 8b | 1.0000 | 0.1701(7) | | 0.1918(8) | 0.75000 | 0.050(5) |
| O(6), 8b | 1.0000 | 0.1706(9) | | 0.4205(7) | 0.75000 | 0.050(5) |
| O(7), 8b | 1.0000 | 0.2254(9) | | 0.50000 | 0.50000 | 0.050(5) |
| O(8), 8b | 1.0000 | 0.25000 | | 0.25000 | 0.50000 | 0.050(5) |
| O(9), 4c | 1.0000 | 0.00000 | | 0.4007(12) | 0.25000 | 0.050(5) |
| O(10), 4c | 1.0000 | 0.00000 | | 0.2029(13) | 0.25000 | 0.050(5) |
| Pb(1), 4c | 0.2570 | 0.50000 | | 0.00000 | 0.00000 | 0.158(7) |
| Pb(2), 4c | 0.1000 | 0.50000 | | 0.088(3) | 0.25000 | 0.158(7) |
| Pb(3), 8b | 0.0525 | 0.50000 | | 0.5858(28) | 0.365(8) | 0.158(7) |
| Pb(4), 8b | 0.2000 | 0.50000 | | 0.3858(9) | 0.0734(25) | 0.158(7) |
| OW(1), 8b | 0.9540 | 0.4347(22) | | 0.2576(17) | 0.25000 | 0.158(7) |
| OW(2), 16a | 0.9234 | 0.4104(10) | | 0.5129(25) | 0.8619(26) | 0.158(7) |

| Pb-MOR (wet) | | | | | | |
| --- | --- | --- | --- | --- | --- | --- |
| Refined unit cell composition | | | Pb_3.2(2)_Al_6.3(5)_Si_41.6(4)_O_96_ ·26.8H_2_O (_w_*R*_p_ 2.084%, χ^2^ 0.74) | | | |
| Refined unit cell parameters | | | *a* 18.112(4) Å, *b* 20.443(4) Å, *c* 7.5019(15) Å, *V* 2777.8(17) Å^3^ | | | |
| Atom, site | Occupancy | x^a^ | | y^a^ | z^a^ | *U_iso_*^a,b^ |
| Si(1), 16a | 0.9906 | 0.1970(5) | | 0.4273(4) | 0.5400(10) | 0.050(5) |
| Si(2), 16a | 0.9906 | 0.1957(4) | | 0.1901(4) | 0.5391(9) | 0.050(5) |
| Si(3), 8b | 0.9906 | 0.0866(5) | | 0.3801(7) | 0.25000 | 0.050(5) |
| Si(4), 8b | 0.9906 | 0.0845(5) | | 0.2224(6) | 0.25000 | 0.050(5) |
| O(1), 16a | 1.0000 | 0.1257(4) | | 0.4089(7) | 0.4270(5) | 0.063(6) |
| O(2), 16a | 1.0000 | 0.1218(3) | | 0.1938(6) | 0.4281(5) | 0.063(6) |
| O(3), 16a | 1.0000 | 0.2643(5) | | 0.37807(31) | 0.5021(21) | 0.063(6) |
| O(4), 8b | 1.0000 | 0.0859(13) | | 0.3013(6) | 0.25000 | 0.063(6) |
| O(5), 8b | 1.0000 | 0.1710(7) | | 0.1911(8) | 0.75000 | 0.063(6) |
| O(6), 8b | 1.0000 | 0.1720(9) | | 0.4214(7) | 0.75000 | 0.063(6) |
| O(7), 8b | 1.0000 | 0.2267(10) | | 0.50000 | 0.50000 | 0.063(6) |
| O(8), 8b | 1.0000 | 0.25000 | | 0.25000 | 0.50000 | 0.063(6) |
| O(9), 4c | 1.0000 | 0.00000 | | 0.4072(11) | 0.25000 | 0.063(6) |
| O(10), 4c | 1.0000 | 0.00000 | | 0.1941(12) | 0.25000 | 0.063(6) |
| Pb(1), 4c | 0.2790 | 0.50000 | | 0.00000 | 0.00000 | 0.178(7) |
| Pb(2), 4c | 0.1623 | 0.50000 | | 0.1150(23) | 0.25000 | 0.178(7) |
| Pb(3), 8b | 0.2161 | 0.50000 | | 0.3901(10) | 0.0751(22) | 0.178(7) |
| OW(1), 8b | 1.0000 | 0.4340(20) | | 0.2768(20) | 0.25000 | 0.178(7) |
| OW(2), 16a | 0.9994 | 0.4194(10) | | 0.4945(17) | 0.8549(30) | 0.178(7) |
| OW(3), 8b | 0.3505 | 0.50000 | | 0.572(4) | 0.322(21) | 0.178(7) |

| Pb-MOR (0.5(1) GPa) | | | | | | |
| --- | --- | --- | --- | --- | --- | --- |
| Refined unit cell composition | | | Pb_3.2(2)_Al_6.3(5)_Si_41.6(4)_O_96_ ·28.9H_2_O (_w_*R*_p_ 2.017%, χ^2^ 0.54) | | | |
| Refined unit cell parameters | | | *a* 18.102(4) Å, *b* 20.388(3) Å, *c* 7.4788(13) Å, *V* 2760.2(14) Å^3^ | | | |
| Atom, site | Occupancy | x^a^ | | y^a^ | z^a^ | *U_iso_*^a,b^ |
| Si(1), 16a | 0.9906 | 0.1932(4) | | 0.42393(31) | 0.5395(9) | 0.041(4) |
| Si(2), 16a | 0.9906 | 0.1951(4) | | 0.1891(3) | 0.5390(8) | 0.041(4) |
| Si(3), 8b | 0.9906 | 0.0800(4) | | 0.3748(6) | 0.25000 | 0.041(4) |
| Si(4), 8b | 0.9906 | 0.0782(4) | | 0.2177(5) | 0.25000 | 0.041(4) |
| O(1), 16a | 1.0000 | 0.1245(4) | | 0.3963(6) | 0.4274(5) | 0.077(6) |
| O(2), 16a | 1.0000 | 0.12032(29) | | 0.1964(5) | 0.4293(5) | 0.077(6) |
| O(3), 16a | 1.0000 | 0.2669(4) | | 0.38046(29) | 0.5077(19) | 0.077(6) |
| O(4), 8b | 1.0000 | 0.0642(8) | | 0.2963(5) | 0.25000 | 0.077(6) |
| O(5), 8b | 1.0000 | 0.1720(6) | | 0.1891(7) | 0.75000 | 0.077(6) |
| O(6), 8b | 1.0000 | 0.1674(8) | | 0.4156(6) | 0.75000 | 0.077(6) |
| O(7), 8b | 1.0000 | 0.2114(9) | | 0.50000 | 0.50000 | 0.077(6) |
| O(8), 8b | 1.0000 | 0.25000 | | 0.25000 | 0.50000 | 0.077(6) |
| O(9), 4c | 1.0000 | 0.00000 | | 0.4146(6) | 0.25000 | 0.077(6) |
| O(10), 4c | 1.0000 | 0.00000 | | 0.1768(7) | 0.25000 | 0.077(6) |
| Pb(1), 4c | 0.2790 | 0.50000 | | 0.00000 | 0.00000 | 0.182(8) |
| Pb(2), 4c | 0.1623 | 0.50000 | | 0.0976(21) | 0.25000 | 0.182(8) |
| Pb(3), 8b | 0.2161 | 0.50000 | | 0.4007(10) | 0.0815(25) | 0.182(8) |
| OW(1), 16a | 0.5478 | 0.4472(19) | | 0.2692(14) | 0.267(22) | 0.182(8) |
| OW(2), 16a | 1.0000 | 0.4217(9) | | 0.4934(15) | 0.825(4) | 0.182(8) |
| OW(3), 8b | 0.5144 | 0.50000 | | 0.542(4) | 0.368(11) | 0.182(8) |

| Pb-MOR (1.2(1) GPa) | | | | | | |
| --- | --- | --- | --- | --- | --- | --- |
| Refined unit cell composition | | | Pb_3.2(2)_Al_6.3(5)_Si_41.6(4)_O_96_ ·32.2H_2_O (_w_*R*_p_ 2.22%, χ^2^ 0.52) | | | |
| Refined unit cell parameters | | | *a* 18.039(6) Å, *b* 20.320(6) Å, *c* 7.4297(22) Å, *V* 2723.4(25) Å^3^ | | | |
| Atom, site | Occupancy | x^a^ | | y^a^ | z^a^ | *U_iso_*^a,b^ |
| Si(1), 16a | 0.9906 | 0.1913(5) | | 0.42368(31) | 0.5426(9) | 0.054(5) |
| Si(2), 16a | 0.9906 | 0.1962(4) | | 0.1881(4) | 0.5364(8) | 0.054(5) |
| Si(3), 8b | 0.9906 | 0.0774(4) | | 0.3740(6) | 0.25000 | 0.054(5) |
| Si(4), 8b | 0.9906 | 0.0766(4) | | 0.2177(6) | 0.25000 | 0.054(5) |
| O(1), 16a | 1.0000 | 0.1249(4) | | 0.3925(6) | 0.4270(4) | 0.092(9) |
| O(2), 16a | 1.0000 | 0.11961(29) | | 0.1987(5) | 0.4313(5) | 0.092(9) |
| O(3), 16a | 1.0000 | 0.2664(4) | | 0.38057(30) | 0.5287(18) | 0.092(9) |
| O(4), 8b | 1.0000 | 0.0576(8) | | 0.2959(5) | 0.25000 | 0.092(9) |
| O(5), 8b | 1.0000 | 0.1774(7) | | 0.1821(7) | 0.75000 | 0.092(9) |
| O(6), 8b | 1.0000 | 0.1588(8) | | 0.4178(6) | 0.75000 | 0.092(9) |
| O(7), 8b | 1.0000 | 0.2085(10) | | 0.50000 | 0.50000 | 0.092(9) |
| O(8), 8b | 1.0000 | 0.25000 | | 0.25000 | 0.50000 | 0.092(9) |
| O(9), 4c | 1.0000 | 0.00000 | | 0.4172(6) | 0.25000 | 0.092(9) |
| O(10), 4c | 1.0000 | 0.00000 | | 0.1736(7) | 0.25000 | 0.092(9) |
| Pb(1), 4c | 0.2790 | 0.50000 | | 0.00000 | 0.00000 | 0.157(8) |
| Pb(2), 4c | 0.1623 | 0.50000 | | 0.1035(23) | 0.25000 | 0.157(8) |
| Pb(3), 8b | 0.2161 | 0.50000 | | 0.4127(12) | 0.0737(31) | 0.157(8) |
| OW(1), 16a | 0.6870 | 0.4423(19) | | 0.2653(12) | 0.282(12) | 0.157(8) |
| OW(2), 16a | 1.0000 | 0.4192(10) | | 0.4922(19) | 0.806(8) | 0.157(8) |
| OW(3), 8b | 0.6502 | 0.50000 | | 0.527(5) | 0.287(13) | 0.157(8) |

| Sr-MOR (ambient) | | | | | | |
| --- | --- | --- | --- | --- | --- | --- |
| Refined unit cell composition | | | Sr_3.3(2)_Al_6.6(2)_Si_41.4(2)_O_96_ ·23H_2_O (_w_*R*_p_ 2.66%, χ^2^ 1.31) | | | |
| Refined unit cell parameters | | | *a* 18.0918(26) Å, *b* 20.4378(28) Å, *c* 7.5065(10) Å, *V* 2775.6(11) Å^3^ | | | |
| Atom, site | Occupancy | x^a^ | | y^a^ | z^a^ | *U_iso_*^a,b^ |
| Si(1), 16a | 0.9900 | 0.2018(5) | | 0.4304(4) | 0.5323(11) | 0.0298(25) |
| Si(2), 16a | 0.9900 | 0.1881(4) | | 0.1894(5) | 0.5426(10) | 0.0298(25) |
| Si(3), 8b | 0.9900 | 0.0893(5) | | 0.3840(6) | 0.25000 | 0.0298(25) |
| Si(4), 8b | 0.9900 | 0.0884(5) | | 0.2237(6) | 0.25000 | 0.0298(25) |
| O(1), 16a | 1.0000 | 0.1268(5) | | 0.4163(6) | 0.4272(7) | 0.035(4) |
| O(2), 16a | 1.0000 | 0.1201(4) | | 0.1904(6) | 0.4270(7) | 0.035(4) |
| O(3), 16a | 1.0000 | 0.2669(5) | | 0.3783(4) | 0.4763(21) | 0.035(4) |
| O(4), 8b | 1.0000 | 0.0940(11) | | 0.3041(6) | 0.25000 | 0.035(4) |
| O(5), 8b | 1.0000 | 0.1616(7) | | 0.1981(8) | 0.75000 | 0.035(4) |
| O(6), 8b | 1.0000 | 0.1841(9) | | 0.4209(8) | 0.75000 | 0.035(4) |
| O(7), 8b | 1.0000 | 0.2363(9) | | 0.50000 | 0.50000 | 0.035(4) |
| O(8), 8b | 1.0000 | 0.25000 | | 0.25000 | 0.50000 | 0.035(4) |
| O(9), 4c | 1.0000 | 0.00000 | | 0.4057(12) | 0.25000 | 0.035(4) |
| O(10), 4c | 1.0000 | 0.00000 | | 0.2034(11) | 0.25000 | 0.035(4) |
| Sr(1), 4c | 0.2000 | 0.00000 | | 0.50000 | 0.00000 | 0.035(7) |
| Sr(2), 8b | 0.2169 | 0.0944(15) | | 0.3965(15) | 0.75000 | 0.035(7) |
| Sr(3), 4c | 0.2000 | 0.00000 | | -0.0457(22) | 0.75000 | 0.035(7) |
| OW(1), 4c | 0.9000 | 0.00000 | | 0.2394(20) | 0.75000 | 0.035(7) |
| OW(2), 16a | 0.7500 | 0.0846(11) | | 0.9996(14) | 0.1410(31) | 0.035(7) |
| OW(3), 8b | 0.9300 | 0.00000 | | 0.1121(14) | -0.088(4) | 0.035(7) |

| Sr-MOR (wet) | | | | | | |
| --- | --- | --- | --- | --- | --- | --- |
| Refined unit cell composition | | | Sr_3.3(2)_Al_6.6(2)_Si_41.4(2)_O_96_ ·26.5H_2_O (_w_*R*_p_ 2.658%, χ^2^ 0.98) | | | |
| Refined unit cell parameters | | | *a* 18.1106(27) Å, *b* 20.4543(30) Å, *c* 7.5061(11) Å, *V* 2780.6(12) Å^3^ | | | |
| Atom, site | Occupancy | x^a^ | | y^a^ | z^a^ | *U_iso_*^a,b^ |
| Si(1), 16a | 0.9900 | 0.2023(4) | | 0.4309(4) | 0.5334(9) | 0.0297(24) |
| Si(2), 16a | 0.9900 | 0.1894(4) | | 0.1899(4) | 0.5422(9) | 0.0297(24) |
| Si(3), 8b | 0.9900 | 0.0881(5) | | 0.3873(6) | 0.25000 | 0.0297(24) |
| Si(4), 8b | 0.9900 | 0.0876(5) | | 0.2269(6) | 0.25000 | 0.0297(24) |
| O(1), 16a | 1.0000 | 0.1271(4) | | 0.4183(5) | 0.4269(6) | 0.037(4) |
| O(2), 16a | 1.0000 | 0.1207(4) | | 0.1933(6) | 0.4259(6) | 0.037(4) |
| O(3), 16a | 1.0000 | 0.2663(5) | | 0.3776(3) | 0.4783(19) | 0.037(4) |
| O(4), 8b | 1.0000 | 0.0917(10) | | 0.3071(5) | 0.25000 | 0.037(4) |
| O(5), 8b | 1.0000 | 0.1619(6) | | 0.1983(7) | 0.75000 | 0.037(4) |
| O(6), 8b | 1.0000 | 0.1841(8) | | 0.4219(7) | 0.75000 | 0.037(4) |
| O(7), 8b | 1.0000 | 0.2384(8) | | 0.50000 | 0.50000 | 0.037(4) |
| O(8), 8b | 1.0000 | 0.25000 | | 0.25000 | 0.50000 | 0.037(4) |
| O(9), 4c | 1.0000 | 0.00000 | | 0.4098(11) | 0.25000 | 0.037(4) |
| O(10), 4c | 1.0000 | 0.00000 | | 0.2052(10) | 0.25000 | 0.037(4) |
| Sr(1), 4c | 0.2176 | 0.00000 | | 0.50000 | 0.00000 | 0.056(7) |
| Sr(2), 8b | 0.2050 | 0.0961(15) | | 0.3931(17) | 0.75000 | 0.056(7) |
| Sr(3), 4c | 0.2000 | 0.00000 | | -0.0336(22) | 0.75000 | 0.056(7) |
| OW(1), 8b | 0.7107 | 0.00000 | | 0.2425(12) | 0.78(11) | 0.056(7) |
| OW(2), 16a | 0.8000 | 0.0851(10) | | 1.0055(13) | 0.1363(28) | 0.056(7) |
| OW(3), 8b | 1.0000 | 0.00000 | | 0.1062(14) | -0.078(4) | 0.056(7) |

| Sr-MOR (0.5(1) GPa) | | | | | | |
| --- | --- | --- | --- | --- | --- | --- |
| Refined unit cell composition | | | Sr_3.3(2)_Al_6.6(2)_Si_41.4(2)_O_96_ ·28.9H_2_O (_w_*R*_p_ 2.75%, χ^2^ 0.85) | | | |
| Refined unit cell parameters | | | *a* 18.077(4) Å, *b* 20.382(4) Å, *c* 7.4788(15) Å, *V* 2755.5(16) Å^3^ | | | |
| Atom, site | Occupancy | x^a^ | | y^a^ | z^a^ | *U_iso_*^a,b^ |
| Si(1), 16a | 0.9900 | 0.2030(5) | | 0.4302(4) | 0.5325(11) | 0.062(4) |
| Si(2), 16a | 0.9900 | 0.1902(5) | | 0.1907(4) | 0.5434(11) | 0.062(4) |
| Si(3), 8b | 0.9900 | 0.0874(5) | | 0.3898(7) | 0.25000 | 0.062(4) |
| Si(4), 8b | 0.9900 | 0.0875(5) | | 0.2285(6) | 0.25000 | 0.062(4) |
| O(1), 16a | 1.0000 | 0.1271(5) | | 0.4191(6) | 0.4277(6) | 0.067(5) |
| O(2), 16a | 1.0000 | 0.1215(4) | | 0.1948(6) | 0.4256(6) | 0.067(5) |
| O(3), 16a | 1.0000 | 0.2663(6) | | 0.3774(3) | 0.4762(21) | 0.067(5) |
| O(4), 8b | 1.0000 | 0.0900(12) | | 0.3089(6) | 0.25000 | 0.067(5) |
| O(5), 8b | 1.0000 | 0.1611(6) | | 0.1998(8) | 0.75000 | 0.067(5) |
| O(6), 8b | 1.0000 | 0.1844(9) | | 0.4209(7) | 0.75000 | 0.067(5) |
| O(7), 8b | 1.0000 | 0.2388(9) | | 0.50000 | 0.50000 | 0.067(5) |
| O(8), 8b | 1.0000 | 0.25000 | | 0.25000 | 0.50000 | 0.067(5) |
| O(9), 4c | 1.0000 | 0.00000 | | 0.4134(12) | 0.25000 | 0.067(5) |
| O(10), 4c | 1.0000 | 0.00000 | | 0.2054(11) | 0.25000 | 0.067(5) |
| Sr(1), 4c | 0.2176 | 0.00000 | | 0.50000 | 0.00000 | 0.068(10) |
| Sr(2), 8b | 0.2050 | 0.0868(19) | | 0.3915(20) | 0.75000 | 0.068(10) |
| Sr(3), 4c | 0.2000 | 0.00000 | | -0.033(3) | 0.75000 | 0.068(10) |
| OW(1), 8b | 0.8640 | 0.00000 | | 0.2449(12) | 0.791(14) | 0.068(10) |
| OW(2), 16a | 0.8716 | 0.0844(10) | | 1.0094(20) | 0.142(3) | 0.068(10) |
| OW(3), 8b | 1.0000 | 0.00000 | | 0.0930(18) | -0.074(5) | 0.068(10) |

| Sr-MOR (1.0(1) GPa) | | | | | | |
| --- | --- | --- | --- | --- | --- | --- |
| Refined unit cell composition | | | Sr_3.3(2)_Al_6.6(2)_Si_41.4(2)_O_96_ ·30.4H_2_O (_w_*R*_p_ 2.683%, χ^2^ 0.76) | | | |
| Refined unit cell parameters | | | *a* 18.063(4) Å, *b* 20.362(4) Å, *c* 7.4644(15) Å, *V* 2745.5(16) Å^3^ | | | |
| Atom, site | Occupancy | x^a^ | | y^a^ | z^a^ | *U_iso_*^a,b^ |
| Si(1), 16a | 0.9900 | 0.2039(4) | | 0.4299(4) | 0.5333(9) | 0.066(4) |
| Si(2), 16a | 0.9900 | 0.1909(4) | | 0.1912(4) | 0.5462(10) | 0.066(4) |
| Si(3), 8b | 0.9900 | 0.0866(5) | | 0.3906(6) | 0.25000 | 0.066(4) |
| Si(4), 8b | 0.9900 | 0.0867(5) | | 0.2291(6) | 0.25000 | 0.066(4) |
| O(1), 16a | 1.0000 | 0.1268(4) | | 0.4195(5) | 0.4286(5) | 0.074(6) |
| O(2), 16a | 1.0000 | 0.1214(4) | | 0.1949(6) | 0.4255(5) | 0.074(6) |
| O(3), 16a | 1.0000 | 0.2663(5) | | 0.37729(30) | 0.4683(18) | 0.074(6) |
| O(4), 8b | 1.0000 | 0.0895(12) | | 0.3095(5) | 0.25000 | 0.074(6) |
| O(5), 8b | 1.0000 | 0.1594(5) | | 0.2025(7) | 0.75000 | 0.074(6) |
| O(6), 8b | 1.0000 | 0.1871(8) | | 0.4190(6) | 0.75000 | 0.074(6) |
| O(7), 8b | 1.0000 | 0.2394(8) | | 0.50000 | 0.50000 | 0.074(6) |
| O(8), 8b | 1.0000 | 0.25000 | | 0.25000 | 0.50000 | 0.074(6) |
| O(9), 4c | 1.0000 | 0.00000 | | 0.4150(11) | 0.25000 | 0.074(6) |
| O(10), 4c | 1.0000 | 0.00000 | | 0.2050(10) | 0.25000 | 0.074(6) |
| Sr(1), 4c | 0.2176 | 0.00000 | | 0.50000 | 0.00000 | 0.086(9) |
| Sr(2), 8b | 0.2050 | 0.0820(19) | | 0.3911(19) | 0.75000 | 0.086(9) |
| Sr(3), 4c | 0.2000 | 0.00000 | | -0.044(3) | 0.75000 | 0.086(9) |
| OW(1), 8b | 0.9769 | 0.00000 | | 0.2447(11) | 0.787(9) | 0.086(9) |
| OW(2), 16a | 0.9107 | 0.0835(9) | | 1.0118(17) | 0.155(4) | 0.086(9) |
| OW(3), 8b | 1.0000 | 0.00000 | | 0.0880(18) | -0.074(5) | 0.086(9) |

| Cd-MOR (ambient) | | | | | | |
| --- | --- | --- | --- | --- | --- | --- |
| Refined unit cell composition | | | Cd_3.4_Al_6.7(1)_Si_41.3(1)_O_96_ ·23H_2_O (_w_*R*_p_ 2.117%, χ^2^ 1.16) | | | |
| Refined unit cell parameters | | | *a* 18.1366(22) Å, *b* 20.3901(25) Å, *c* 7.4964(9) Å, *V* 2772.2(10) Å^3^ | | | |
| Atom, site | Occupancy | x^a^ | | y^a^ | z^a^ | *U_iso_*^a,b^ |
| Si(1), 16a | 0.9900 | 0.2023(4) | | 0.4291(4) | 0.5336(10) | 0.0194(20) |
| Si(2), 16a | 0.9900 | 0.1893(4) | | 0.1909(4) | 0.5398(9) | 0.0194(20) |
| Si(3), 8b | 0.9900 | 0.0851(5) | | 0.3877(5) | 0.25000 | 0.0194(20) |
| Si(4), 8b | 0.9900 | 0.0859(5) | | 0.2235(5) | 0.25000 | 0.0194(20) |
| O(1), 16a | 1.0000 | 0.1299(5) | | 0.4153(5) | 0.4233(14) | 0.040(3) |
| O(2), 16a | 1.0000 | 0.1212(4) | | 0.1958(6) | 0.4285(6) | 0.040(3) |
| O(3), 16a | 1.0000 | 0.2667(5) | | 0.3769(3) | 0.4952(21) | 0.040(3) |
| O(4), 8b | 1.0000 | 0.0826(9) | | 0.3059(5) | 0.25000 | 0.040(3) |
| O(5), 8b | 1.0000 | 0.1635(7) | | 0.1951(8) | 0.75000 | 0.040(3) |
| O(6), 8b | 1.0000 | 0.1782(9) | | 0.4248(7) | 0.75000 | 0.040(3) |
| O(7), 8b | 1.0000 | 0.2391(8) | | 0.50000 | 0.50000 | 0.040(3) |
| O(8), 8b | 1.0000 | 0.25000 | | 0.25000 | 0.50000 | 0.040(3) |
| O(9), 4c | 1.0000 | 0.00000 | | 0.4147(9) | 0.25000 | 0.040(3) |
| O(10), 4c | 1.0000 | 0.00000 | | 0.1963(9) | 0.25000 | 0.040(3) |
| Cd(1), 4c | 0.1425 | 0.00000 | | 0.50000 | 0.00000 | 0.104(11) |
| Cd(2), 8b | 0.1956 | 0.0596(13) | | 0.3895(11) | 0.75000 | 0.104(11) |
| Cd(3), 8b | 0.1531 | 0.00000 | | 0.1312(13) | 0.705(7) | 0.104(11) |
| OW(1), 4c | 0.6000 | 0.00000 | | 0.236(4) | 0.75000 | 0.104(11) |
| OW(2), 16a | 0.9400 | 0.0771(7) | | 1.0130(10) | 0.1442(25) | 0.104(11) |
| OW(3), 8b | 0.7000 | 0.00000 | | 0.0917(13) | 0.997(6) | 0.104(11) |

| Cd-MOR (wet) | | | | | | |
| --- | --- | --- | --- | --- | --- | --- |
| Refined unit cell composition | | | Cd_3.4_Al_6.7(1)_Si_41.3(1)_O_96_ ·25.7H_2_O (_w_*R*_p_ 1.713%, χ^2^ 0.77) | | | |
| Refined unit cell parameters | | | *a* 18.1576(25) Å, *b* 20.4338(28) Å, *c* 7.5015(10) Å, *V* 2783.3(11) Å^3^ | | | |
| Atom, site | Occupancy | x^a^ | | y^a^ | z^a^ | *U_iso_*^a,b^ |
| Si(1), 16a | 0.9900 | 0.2039(4) | | 0.4290(3) | 0.5335(9) | 0.0104(18) |
| Si(2), 16a | 0.9900 | 0.1903(4) | | 0.1907(4) | 0.5400(9) | 0.0104(18) |
| Si(3), 8b | 0.9900 | 0.0858(4) | | 0.3862(5) | 0.25000 | 0.0104(18) |
| Si(4), 8b | 0.9900 | 0.0838(4) | | 0.2240(5) | 0.25000 | 0.0104(18) |
| O(1), 16a | 1.0000 | 0.1292(4) | | 0.4134(5) | 0.4269(5) | 0.051(4) |
| O(2), 16a | 1.0000 | 0.1209(3) | | 0.1951(5) | 0.4265(5) | 0.051(4) |
| O(3), 16a | 1.0000 | 0.2677(5) | | 0.37757(29) | 0.4877(19) | 0.051(4) |
| O(4), 8b | 1.0000 | 0.0830(9) | | 0.3049(5) | 0.25000 | 0.051(4) |
| O(5), 8b | 1.0000 | 0.1637(6) | | 0.1965(7) | 0.75000 | 0.051(4) |
| O(6), 8b | 1.0000 | 0.1831(8) | | 0.4223(7) | 0.75000 | 0.051(4) |
| O(7), 8b | 1.0000 | 0.2381(7) | | 0.50000 | 0.50000 | 0.051(4) |
| O(8), 8b | 1.0000 | 0.25000 | | 0.25000 | 0.50000 | 0.051(4) |
| O(9), 4c | 1.0000 | 0.00000 | | 0.4123(9) | 0.25000 | 0.051(4) |
| O(10), 4c | 1.0000 | 0.00000 | | 0.1957(8) | 0.25000 | 0.051(4) |
| Cd(1), 4c | 0.1425 | 0.00000 | | 0.50000 | 0.00000 | 0.122(16) |
| Cd(2), 8b | 0.1956 | 0.0630(15) | | 0.3982(13) | 0.75000 | 0.122(16) |
| Cd(3), 8b | 0.1531 | 0.00000 | | 0.1469(12) | 0.748(10) | 0.122(16) |
| OW(1), 4c | 0.7514 | 0.00000 | | 0.281(4) | 0.75000 | 0.122(16) |
| OW(2), 16a | 1.0000 | 0.0696(7) | | 1.0040(12) | 0.174(4) | 0.122(16) |
| OW(3), 8b | 0.8400 | 0.00000 | | 0.1024(11) | 0.985(5) | 0.122(16) |

| Cd-MOR (0.5(1) GPa) | | | | | | |
| --- | --- | --- | --- | --- | --- | --- |
| Refined unit cell composition | | | Cd_3.4_Al_6.7(1)_Si_41.3(1)_O_96_ ·26.5H_2_O (_w_*R*_p_ 2.102%, χ^2^ 1.12) | | | |
| Refined unit cell parameters | | | *a* 18.117(3) Å, *b* 20.394(4) Å, *c* 7.4802(13) Å, *V* 2763.8(14) Å^3^ | | | |
| Atom, site | Occupancy | x^a^ | | y^a^ | z^a^ | *U_iso_*^a,b^ |
| Si(1), 16a | 0.9900 | 0.2022(5) | | 0.4276(4) | 0.5351(11) | 0.0296(26) |
| Si(2), 16a | 0.9900 | 0.1914(4) | | 0.1912(4) | 0.5409(10) | 0.0296(26) |
| Si(3), 8b | 0.9900 | 0.0854(5) | | 0.3862(6) | 0.25000 | 0.0296(26) |
| Si(4), 8b | 0.9900 | 0.0848(5) | | 0.2238(6) | 0.25000 | 0.0296(26) |
| O(1), 16a | 1.0000 | 0.1283(5) | | 0.4124(6) | 0.4278(6) | 0.066(5) |
| O(2), 16a | 1.0000 | 0.1219(4) | | 0.1955(6) | 0.4274(6) | 0.066(5) |
| O(3), 16a | 1.0000 | 0.2678(5) | | 0.3778(4) | 0.4916(23) | 0.066(5) |
| O(4), 8b | 1.0000 | 0.0825(11) | | 0.3048(6) | 0.25000 | 0.066(5) |
| O(5), 8b | 1.0000 | 0.1651(7) | | 0.1954(8) | 0.75000 | 0.066(5) |
| O(6), 8b | 1.0000 | 0.1800(9) | | 0.4212(8) | 0.75000 | 0.066(5) |
| O(7), 8b | 1.0000 | 0.2348(9) | | 0.50000 | 0.50000 | 0.066(5) |
| O(8), 8b | 1.0000 | 0.25000 | | 0.25000 | 0.50000 | 0.066(5) |
| O(9), 4c | 1.0000 | 0.00000 | | 0.4139(10) | 0.25000 | 0.066(5) |
| O(10), 4c | 1.0000 | 0.00000 | | 0.1946(10) | 0.25000 | 0.066(5) |
| Cd(1), 4c | 0.1425 | 0.00000 | | 0.50000 | 0.00000 | 0.156(20) |
| Cd(2), 8b | 0.1956 | 0.0539(16) | | 0.3991(14) | 0.75000 | 0.156(20) |
| Cd(3), 8b | 0.1531 | 0.00000 | | 0.1452(15) | 0.788(8) | 0.156(20) |
| OW(1), 4c | 0.8470 | 0.00000 | | 0.269(4) | 0.75000 | 0.156(20) |
| OW(2), 16a | 1.0000 | 0.0679(8) | | 1.0018(17) | 0.188(7) | 0.156(20) |
| OW(3), 8b | 0.8841 | 0.00000 | | 0.0663(20) | 1.037(10) | 0.156(20) |

| Cd-MOR (1.0(1) GPa) | | | | | | |
| --- | --- | --- | --- | --- | --- | --- |
| Refined unit cell composition | | | Cd_3.4_Al_6.7(1)_Si_41.3(1)_O_96_ ·28H_2_O (_w_*R*_p_ 2.410%, χ^2^ 1.59) | | | |
| Refined unit cell parameters | | | *a* 18.0584(19) Å, *b* 20.3578(18) Å, *c* 7.4487(6) Å, *V* 2738.4(7) Å^3^ | | | |
| Atom, site | Occupancy | x^a^ | | y^a^ | z^a^ | *U_iso_*^a,b^ |
| Si(1), 16a | 0.9900 | 0.2034(5) | | 0.4271(4) | 0.5355(12) | 0.0522(27) |
| Si(2), 16a | 0.9900 | 0.1921(5) | | 0.1914(5) | 0.5401(12) | 0.0522(27) |
| Si(3), 8b | 0.9900 | 0.0850(6) | | 0.3863(7) | 0.25000 | 0.0522(27) |
| Si(4), 8b | 0.9900 | 0.0847(6) | | 0.2236(7) | 0.25000 | 0.0522(27) |
| O(1), 16a | 1.0000 | 0.1282(5) | | 0.4117(7) | 0.4289(8) | 0.098(5) |
| O(2), 16a | 1.0000 | 0.1220(5) | | 0.1950(7) | 0.4279(8) | 0.098(5) |
| O(3), 16a | 1.0000 | 0.2690(6) | | 0.3783(4) | 0.4897(26) | 0.098(5) |
| O(4), 8b | 1.0000 | 0.0828(12) | | 0.3045(6) | 0.25000 | 0.098(5) |
| O(5), 8b | 1.0000 | 0.1638(8) | | 0.1971(10) | 0.75000 | 0.098(5) |
| O(6), 8b | 1.0000 | 0.1798(11) | | 0.4196(9) | 0.75000 | 0.098(5) |
| O(7), 8b | 1.0000 | 0.2336(10) | | 0.50000 | 0.50000 | 0.098(5) |
| O(8), 8b | 1.0000 | 0.25000 | | 0.25000 | 0.50000 | 0.098(5) |
| O(9), 4c | 1.0000 | 0.00000 | | 0.4146(11) | 0.25000 | 0.098(5) |
| O(10), 4c | 1.0000 | 0.00000 | | 0.1931(11) | 0.25000 | 0.098(5) |
| Cd(1), 4c | 0.1425 | 0.00000 | | 0.50000 | 0.00000 | 0.188(19) |
| Cd(2), 8b | 0.1956 | 0.0486(17) | | 0.4042(14) | 0.75000 | 0.188(19) |
| Cd(3), 8b | 0.1531 | 0.00000 | | 0.1489(15) | 0.786(10) | 0.188(19) |
| OW(1), 4c | 1.0000 | 0.00000 | | 0.285(3) | 0.75000 | 0.188(19) |
| OW(2), 16a | 1.0000 | 0.0648(9) | | 0.9992(17) | 0.216(25) | 0.188(19) |
| OW(3), 8b | 1.0000 | 0.00000 | | 0.0589(18) | 1.039(9) | 0.188(19) |

| EuNa-MOR (ambient) | | | | | | |
| --- | --- | --- | --- | --- | --- | --- |
| Refined unit cell composition | | | Eu_2_Na_0.9_Al_6.4(4)_Si_41.6(4)_O_96_ ·29.6H_2_O (_w_*R*_p_ 2.202%, χ^2^ 2.45) | | | |
| Refined unit cell parameters | | | *a* 18.1064(21) Å, *b* 20.3731(25) Å, *c* 7.4955(9) Å, *V* 2765.0(9) Å^3^ | | | |
| Atom, site | Occupancy | x^a^ | | y^a^ | z^a^ | *U_iso_*^a,b^ |
| Si(1), 16a | 0.9902 | 0.1979(5) | | 0.4270(5) | 0.5326(11) | 0.0251(19) |
| Si(2), 16a | 0.9902 | 0.1932(4) | | 0.1904(5) | 0.5379(11) | 0.0251(19) |
| Si(3), 8b | 0.9902 | 0.0880(6) | | 0.3880(7) | 0.25000 | 0.0251(19) |
| Si(4), 8b | 0.9902 | 0.0960(6) | | 0.2275(7) | 0.25000 | 0.0251(19) |
| O(1), 16a | 1.0000 | 0.1243(6) | | 0.4126(7) | 0.4307(9) | 0.014(3) |
| O(2), 16a | 1.0000 | 0.1259(5) | | 0.1968(7) | 0.4300(8) | 0.014(3) |
| O(3), 16a | 1.0000 | 0.2642(6) | | 0.3770(5) | 0.4873(21) | 0.014(3) |
| O(4), 8b | 1.0000 | 0.0944(10) | | 0.3073(6) | 0.25000 | 0.014(3) |
| O(5), 8b | 1.0000 | 0.1581(7) | | 0.2012(10) | 0.75000 | 0.014(3) |
| O(6), 8b | 1.0000 | 0.1663(10) | | 0.4167(8) | 0.75000 | 0.014(3) |
| O(7), 8b | 1.0000 | 0.2220(10) | | 0.50000 | 0.50000 | 0.014(3) |
| O(8), 8b | 1.0000 | 0.25000 | | 0.25000 | 0.50000 | 0.014(3) |
| O(9), 4c | 1.0000 | 0.00000 | | 0.4102(12) | 0.25000 | 0.014(3) |
| O(10), 4c | 1.0000 | 0.00000 | | 0.2049(12) | 0.25000 | 0.014(3) |
| Eu(1), 8b | 0.0694 | 0.50000 | | 0.0831(22) | 3.28(8) | 0.214(10) |
| Eu(2), 4c | 0.3667 | 0.00000 | | 1.0080(15) | 0.75000 | 0.214(10) |
| Na(1), 8b | 0.1100 | 0.083(11) | | 0.391(8) | 0.75000 | 0.214(10) |
| OW(1), 4c | 1.0000 | 0.00000 | | 0.2988(23) | 0.75000 | 0.214(10) |
| OW(2), 16a | 1.0000 | 0.1011(9) | | 0.0115(21) | 0.0706(26) | 0.214(10) |
| OW(3), 16a | 0.6000 | -0.016(6) | | 0.1098(21) | -0.107(3) | 0.214(10) |

| EuNa-MOR (wet) | | | | | | |
| --- | --- | --- | --- | --- | --- | --- |
| Refined unit cell composition | | | Eu_2_Na_0.9_Al_6.4(4)_Si_41.6(4)_O_96_ ·30.8H_2_O (_w_*R*_p_ 2.135%, χ^2^ 1.24) | | | |
| Refined unit cell parameters | | | *a* 18.0966(18) Å, *b* 20.3509(22) Å, *c* 7.4787(7) Å, *V* 2754.3(8) Å^3^ | | | |
| Atom, site | Occupancy | x^a^ | | y^a^ | z^a^ | *U_iso_*^a,b^ |
| Si(1), 16a | 0.9902 | 0.1983(5) | | 0.4246(4) | 0.5348(11) | 0.0317(22 |
| Si(2), 16a | 0.9902 | 0.1922(4) | | 0.1914(4) | 0.5386(10) | 0.0317(22 |
| Si(3), 8b | 0.9902 | 0.0875(5) | | 0.3913(6) | 0.25000 | 0.0317(22 |
| Si(4), 8b | 0.9902 | 0.0929(5) | | 0.2303(6) | 0.25000 | 0.0317(22 |
| O(1), 16a | 1.0000 | 0.1248(5) | | 0.4156(6) | 0.4311(7) | 0.043(4) |
| O(2), 16a | 1.0000 | 0.1249(4) | | 0.1984(6) | 0.4274(7) | 0.043(4) |
| O(3), 16a | 1.0000 | 0.2646(5) | | 0.3768(4) | 0.4857(19) | 0.043(4) |
| O(4), 8b | 1.0000 | 0.0875(10) | | 0.3099(5) | 0.25000 | 0.043(4) |
| O(5), 8b | 1.0000 | 0.1626(6) | | 0.1989(8) | 0.75000 | 0.043(4) |
| O(6), 8b | 1.0000 | 0.1745(9) | | 0.4176(7) | 0.75000 | 0.043(4) |
| O(7), 8b | 1.0000 | 0.2281(9) | | 0.50000 | 0.50000 | 0.043(4) |
| O(8), 8b | 1.0000 | 0.25000 | | 0.25000 | 0.50000 | 0.043(4) |
| O(9), 4c | 1.0000 | 0.00000 | | 0.4181(10) | 0.25000 | 0.043(4) |
| O(10), 4c | 1.0000 | 0.00000 | | 0.2047(9) | 0.25000 | 0.043(4) |
| Eu(1), 8b | 0.0694 | 0.50000 | | 0.0786(32) | 7.245(12) | 0.227(14) |
| Eu(2), 4c | 0.3667 | 0.00000 | | 0.9995(16) | 0.75000 | 0.227(14) |
| Na(1), 8b | 0.1100 | 0.108(18) | | 0.375(16) | 0.75000 | 0.227(14) |
| OW(1), 8b | 0.76(3) | 0.00000 | | 0.3227(31) | -2.158(8) | 0.227(14) |
| OW(2), 16a | 1.0000 | 0.0880(8) | | 0.0007(26) | 0.123(4) | 0.227(14) |
| OW(3), 16a | 0.542(20) | -0.012(9) | | 0.1302(13) | -0.157(6) | 0.227(14) |

| EuNa-MOR (0.5(1) GPa) | | | | | | |
| --- | --- | --- | --- | --- | --- | --- |
| Refined unit cell composition | | | Eu_2_Na_0.9_Al_6.4(4)_Si_41.6(4)_O_96_ ·31.5H_2_O (_w_*R*_p_ 2.212%, χ^2^ 1.3) | | | |
| Refined unit cell parameters | | | *a* 18.0459(20) Å, *b* 20.3127(23) Å, *c* 7.4573(8) Å, *V* 2733.6(9) Å^3^ | | | |
| Atom, site | Occupancy | x^a^ | | y^a^ | z^a^ | *U_iso_*^a,b^ |
| Si(1), 16a | 0.9902 | 0.1996(5) | | 0.4245(4) | 0.5359(10) | 0.0297(25) |
| Si(2), 16a | 0.9902 | 0.1920(4) | | 0.1920(4) | 0.5378(10) | 0.0297(25) |
| Si(3), 8b | 0.9902 | 0.0879(5) | | 0.3939(6) | 0.25000 | 0.0297(25) |
| Si(4), 8b | 0.9902 | 0.0917(5) | | 0.2335(6) | 0.25000 | 0.0297(25) |
| O(1), 16a | 1.0000 | 0.1261(5) | | 0.4157(6) | 0.4323(7) | 0.048(4) |
| O(2), 16a | 1.0000 | 0.1238(4) | | 0.2015(6) | 0.4281(7) | 0.048(4) |
| O(3), 16a | 1.0000 | 0.2671(5) | | 0.3769(4) | 0.4842(20) | 0.048(4) |
| O(4), 8b | 1.0000 | 0.0845(10) | | 0.3124(5) | 0.25000 | 0.048(4) |
| O(5), 8b | 1.0000 | 0.1617(6) | | 0.2009(8) | 0.75000 | 0.048(4) |
| O(6), 8b | 1.0000 | 0.1773(9) | | 0.4166(7) | 0.75000 | 0.048(4) |
| O(7), 8b | 1.0000 | 0.2297(9) | | 0.50000 | 0.50000 | 0.048(4) |
| O(8), 8b | 1.0000 | 0.25000 | | 0.25000 | 0.50000 | 0.048(4) |
| O(9), 4c | 1.0000 | 0.00000 | | 0.4228(10) | 0.25000 | 0.048(4) |
| O(10), 4c | 1.0000 | 0.00000 | | 0.2043(9) | 0.25000 | 0.048(4) |
| Eu(1), 8b | 0.0694 | 0.50000 | | 0.0710(27) | 7.24(13) | 0.226(13) |
| Eu(2), 4c | 0.3667 | 0.00000 | | 0.9984(16) | 0.75000 | 0.226(13) |
| Na(1), 8b | 0.1100 | 0.097(15) | | 0.375(15) | 0.75000 | 0.226(13) |
| OW(1), 8b | 0.8519 | 0.00000 | | 0.3215(22) | -2.159(6) | 0.226(13) |
| OW(2), 16a | 1.0000 | 0.0870(8) | | 0.0022(23) | 0.127(5) | 0.226(13) |
| OW(3), 16a | 0.5418 | 0.014(6) | | 0.1307(13) | -0.155(5) | 0.226(13) |

| EuNa-MOR (1.0(1) GPa) | | | | | | |
| --- | --- | --- | --- | --- | --- | --- |
| Refined unit cell composition | | | Eu_2_Na_0.9_Al_6.4(4)_Si_41.6(4)_O_96_ ·32.4H_2_O (_w_*R*_p_ 2.296%, χ^2^ 1.38) | | | |
| Refined unit cell parameters | | | *a* 17.9877(24) Å, *b* 20.2876(28) Å, *c* 7.4355(10) Å, *V* 2713.4(10) Å^3^ | | | |
| Atom, multiplicity | Occupancy | x^a^ | | y^a^ | z^a^ | *U_iso_*^a,b^ |
| Si(1), 16a | 0.9902 | 0.2000(5) | | 0.4252(4) | 0.5377(11) | 0.0293(28) |
| Si(2), 16a | 0.9902 | 0.1920(4) | | 0.1922(5) | 0.5368(12) | 0.0293(28) |
| Si(3), 8b | 0.9902 | 0.0887(6) | | 0.3938(6) | 0.25000 | 0.0293(28) |
| Si(4), 8b | 0.9902 | 0.0924(6) | | 0.2332(6) | 0.25000 | 0.0293(28) |
| O(1), 16a | 1.0000 | 0.1254(5) | | 0.4181(6) | 0.4329(7) | 0.048(5) |
| O(2), 16a | 1.0000 | 0.1239(4) | | 0.1988(6) | 0.4273(7) | 0.048(5) |
| O(3), 16a | 1.0000 | 0.2657(6) | | 0.3769(4) | 0.4765(20) | 0.048(5) |
| O(4), 8b | 1.0000 | 0.0880(12) | | 0.3120(5) | 0.25000 | 0.048(5) |
| O(5), 8b | 1.0000 | 0.1601(7) | | 0.2024(9) | 0.75000 | 0.048(5) |
| O(6), 8b | 1.0000 | 0.1791(10) | | 0.4169(8) | 0.75000 | 0.048(5) |
| O(7), 8b | 1.0000 | 0.2323(10) | | 0.50000 | 0.50000 | 0.048(5) |
| O(8), 8b | 1.0000 | 0.25000 | | 0.25000 | 0.50000 | 0.048(5) |
| O(9), 4c | 1.0000 | 0.00000 | | 0.4207(11) | 0.25000 | 0.048(5) |
| O(10), 4c | 1.0000 | 0.00000 | | 0.2055(11) | 0.25000 | 0.048(5) |
| Eu(1), 8b | 0.0694 | 0.50000 | | 0.0701(31) | 7.28(9) | 0.224(15) |
| Eu(2), 4c | 0.3667 | 0.00000 | | 0.9983(18) | 0.75000 | 0.224(15) |
| Na(1), 8b | 0.1100 | 0.098(17) | | 0.374(16) | 0.75000 | 0.224(15) |
| OW(1), 8b | 0.9443 | 0.00000 | | 0.3149(23) | -2.150(6) | 0.224(15) |
| OW(2), 16a | 1.0000 | 0.0869(9) | | 0.0020(26) | 0.127(5) | 0.224(15) |
| OW(3), 16a | 0.5504 | -0.00(3) | | 0.1328(14) | -0.154(6) | 0.224(15) |

^a^Esd’s are in parentheses.

^b^Isotropic displacement factors (*U*_iso_) were refined by grouping the framework tetrahedral atoms, the framework oxygen atoms, the non-framework contents, respectively.
